# Research on evolutionary game of environmental accounting information disclosure from the perspective of multi-agent

**DOI:** 10.1371/journal.pone.0256046

**Published:** 2021-08-31

**Authors:** Yi’ang Qi, Jingjing Yao, Lindong Liu

**Affiliations:** 1 School of Finance and Economics, Jiangsu University, Zhenjiang, Jiangsu, China; 2 School of Mathematical Sciences, Jiangsu University, Zhenjiang, Jiangsu, China; China University of Mining and Technology, CHINA

## Abstract

In the past, China, like other developing countries in the world, pursued the goal of rapid economic development at the expense of ecology and ignored the issue of environmental protection. But in recent decades, as environmental problems have become increasingly prominent, developing countries have begun to explore ways to coordinate economy and ecological environment. As the largest developing country, China has been actively exploring ecological governance plans, putting forward the concept of green development, setting the goal of building a "beautiful China" and placing the construction of ecological civilization in the ontological status of social systems and national goals. In order to accelerate the green development process of enterprises in developing countries, based on the actual situation in China, this paper constructs a tripartite evolutionary game model of environmental accounting information disclosure with enterprises, investors and media as the research objects, and analyzes the internal mechanism of environmental accounting information disclosure. The model finds that the equilibrium of the three parties is affected by multiple factors. Therefore, this study further uses system dynamics to explore the dynamic process of evolutionary games and the strategic choices among multiple agents, and explore the mechanism of three types of agents to promote environmental accounting information disclosure. The simulation results reveal that government incentives have a greater impact on guiding enterprises and the media to evolve in the direction of legal disclosure and participation in exposure strategies. In addition, the continuous reduction of the cumbersome degree of disclosure procedures and the difficulty of improving environmental performance can fundamentally promote companies to actively choose legal disclosure strategies, which will help improve the quality of environmental accounting information disclosure, boost investor confidence, and enhance government supervision.

## Introduction

With the acceleration of the global industrialization process, the greenhouse effect has also intensified. Every country is facing the challenge of sustainable development. However, developing countries have lagged behind developed countries in terms of theory and policy, as well as in specific practices. In many developing countries, the government does not expressly stipulate the disclosure requirements of enterprises for environmental accounting information, and the enterprises themselves have very weak awareness of environmental responsibility. As the largest developing country in the world, China has experienced rapid economic development in recent decades, but there are still many problems in environmental protection. Taking China as the research object, this paper explores the factors that affect the quality of China’s environmental accounting information disclosure and the game mechanism among multiple parties, so as to provide feasible suggestions for the development of environmental protection, energy conservation, emission reduction and green economy in China and other developing countries. The Fifth Plenary Session of the 19th Central Committee proposed that it is necessary to accelerate the promotion of green and low-carbon development, continue to improve the quality of the environment and the stability of the ecosystem, and comprehensively improve the efficiency of resource utilization. In order to promote the sustainable development of the Yangtze River Economic Belt and steadily promote the "ecological priority and green development", in the process of achieving "green transformation", environmental accounting information disclosure has been paid more attention as a requirement of Chinese macro management.

In the 1970s, western scholars began to pay attention to the environmental pollution caused by the production and operation of enterprises, and thus put forward the concept of environmental accounting. Since the 1990s, various international organizations and western countries have reached a consensus on sustainable development in order to protect the environment and reduce pollution, and they have begun to pay attention to the research of environmental accounting, and the environmental accounting information disclosure system has gradually developed. However, China began its preliminary research on environmental accounting in the 1990s. Compared with European and American countries, China’s related research started later, the development time was shorter and the progress was slower, and the research direction also focused more on qualitative disclosure of information. The current research on environmental accounting information disclosure mainly focuses on the disclosure mode, disclosure content, disclosure subject, etc. For the research of environmental accounting information disclosure content, Cormier and Gordon. [1] believed that environmental accounting information includes waste recycling, site greening and environmental governance costs. Greg Trompeter and Patten [2] analyzed the environmental information in the annual reports of listed companies and believed that environmental laws and regulations, environmental risks and corresponding strategies, and environmental pollution control should be further subdivided. Walter Alerts, et al. [3] proposed that environmental accounting information should increase the company’s sustainable development status, environmental governance expenditures, and the company’s compliance with environmental laws. Patrick de Beer and Friend [4] believed that companies prefer to combine the disclosure of environmental issues with the disclosure of the company’s favorable conditions. Dennis Patten and Trompeter [2] believed that the risk sub-categories should be further subdivided after studying environmental information. Emilia Vasile [5] believed that environmental accounting is a collection of multiple information resources, and further expanded the definition of environmental accounting.

In addition, for the study of disclosure mode, Freedman, et al. [6] conducted research on manufacturing companies in Canada and the United States and believed that independent reporting is the preferred method of disclosure for most companies. Holland, et al. [7] found that most American companies disclosed environmental accounting information dispersedly in sustainability reports and social responsibility reports, while European companies chose to focus on the environmental module for disclosure. Jenkins and Yakovleva [8] conducted research on ten leading mining companies and found that their disclosure models and disclosure contents are different. Frost believed that the level of disclosure in independent reports is higher than the level disclosed in the website and annual reports. With the increase of independent reports and websites and other disclosure modes, the number of companies using annual reports to disclose environmental information has decreased [9]. Aerts and Cormier [10] found that the disclosure of environmental information by companies through different channels is also significantly different. Clarkson [11] pointed out that companies that disclose environmental information through independent reports and company websites have relatively high voluntariness and accuracy.

In the process of studying corporate environmental accounting information disclosure behavior, some scholars pointed out that multiple parties are involved in the disclosure process, and found that there is a game relationship between corporate and regulatory agencies or the public or third-party audits. But few studies put the three stakeholders together to analyze the internal game relationship. Bento AM, et al. [12] built a game decision model under the rigid constraints of carbon emission reduction in corporate environmental accounting information. They introduced external conditions such as the government and the public, and analyzed the company’s carbon emissions in combination with internal management. The cost of abatement and its overall emissions are required to be disclosed in the notes. Lohmann L.A [13] considered the impact on corporate environmental accounting information disclosure from the perspective of government supervision. Through research on the relevance of listed company reports, it is concluded that the government’s strict supervision mechanism can effectively promote the release of higher-quality environmental reports by enterprises, and can also enhance the self-consciousness of enterprises to truthfully disclose environmental accounting information. However, it failed to integrate with environmental auditing. Michael Vardon, et al. [14] believe that the government and enterprises cannot make correct decisions without the relevant information provided by the environmental economic accounting system.

With the rise of environmental accounting, more and more listed companies pay attention to environmental accounting information disclosure. The environmental information disclosure process involves issues such as environmental governance fees, disclosure costs, and fines from relevant departments. During the disclosure process, many stakeholders are involved, mainly investors, media, listed companies, and governments. Many listed companies are aware of the importance of protecting the environment, but in order to obtain more corporate benefits, the initiative to disclose environmental accounting information will be weakened, and they will also choose to avoid it during the entire disclosure process. In the process of investment, investors often have vague judgments on the environmental accounting information disclosed by listed companies due to reasons such as academic qualifications, investment experience, and access to investment information. In the process of external reporting of relevant information, the media will also consider a series of issues such as whether the disclosed information can attract public interest, gain continuous attention, and report costs. Therefore, this article introduces the media as one of the main subjects on the basis of the previous scholars’ research, constructs a tripartite evolutionary game model of enterprises, investors and media, and uses system dynamics to analyze the evolution mechanism that affects environmental accounting information disclosure, which is helpful to clarify the game relationship between enterprises, investors and media. To a certain extent, this paper can further supplement the research on the relationship between stakeholders in the field of environmental accounting information disclosure, and propose countermeasures for the efficiency of environmental information disclosure.

This paper is organized as follows. After the introduction, Section 2 provides a literature review. Section 3 introduces the method adopted by the model. The results are analyzed and discussed in Section 4. Finally, a conclusion of the paper and policy implications is provided in Section 5.

## Literature review

Scholars in China and abroad have conducted extensive research in the fields of environmental accounting and information disclosure. The research on environmental accounting information disclosure quality has mainly focused on corporate governance and external influence factors. This paper focuses on the study of the internal mechanism that affects corporate environmental accounting information disclosure. Hermann [15] proposed that the disclosure of environmental accounting information is to promote the sustainable development of enterprises. Patrick de Beer [4] proposed that environmental accounting information disclosure is a tool to measure the level of corporate environmental and economic management, and it is an active disclosure by companies to prove their management level to stakeholders. Pan Ane [16] pointed out that the current problems when companies disclose environmental accounting information mainly include incomplete disclosure, too single disclosure mode, and low disclosure efficiency. Stanwick, et al. [17] believed that to improve the environmental information disclosure system and improve the quality of environmental information disclosure, it is necessary to increase the participation of the public. They recommended the establishment of a set of information disclosure evaluation index system and supervision and management mechanism. In addition, the environmental information disclosed by a company will more or less affect its own business conditions, and even affect the survival and development of the company. Habiba Al-Shaer [18] pointed out that the quantity and quality of corporate environmental accounting information disclosure will significantly affect the perception of external users of the company’s stock.

For the research on the relationship between corporate governance and environmental accounting information disclosure quality, shareholder theory believed that managers disclose environmental information to meet the needs of shareholders, and it considers the relationship between enterprises and shareholders. Forker [19] used the voluntary information disclosure data of large Canadian listed companies and found that the level of corporate governance and the quality of environmental accounting information disclosure are significantly related. In terms of corporate governance structure, if the board of directors has more external directors, it can encourage the company to voluntarily disclose more environmental information, indicating that the corporate governance structure is positively related to environmental information disclosure, but it’s not a significant factor. Besides, a highly dispersed equity distribution is not conducive to the improvement of the quality of accounting information disclosure.

For the study of external factors, Deegan and Rankin [20] conducted a study on 20 Australian companies accused of violating environmental protection laws, which showed that when companies are subject to litigation by regulatory authorities for violating environmental regulations, companies will increase environmental accounting information disclosure, especially positive ones, to reduce or eliminate this regulatory threat. Peatten.D.M [21] believed that only when companies are bound by relevant rules and regulations will they disclose environmental accounting information. Cho [22] believed that the disclosure of corporate environmental accounting information is a function of the public pressure that a company bears in the social and political environment, and this public pressure comes from the cultural environment, political environment, and legal environment. Studies by Barth, Hughes, et al. and Alciatore, et al. [23] have all confirmed that the FASB, SEC and other regulatory agencies’ environmental-related regulations have a substantial impact on environmental information disclosure.

By reviewing and combing the related literature on environmental accounting information disclosure, it can be known that at the beginning of the last century, developed countries have begun theoretical research and practical exploration of environmental accounting, which started very early and carried out comprehensively in the next few decades. So far, a complete set of environmental accounting theoretical systems has been formed from environmental accounting information disclosure to environmental performance, environmental auditing, etc. In addition, the national government has given comprehensive legal protection to the research and practice in this area, so as to effectively supervise and manage the company’s environmental accounting information disclosure. Whether it is theoretical research or practical exploration, developed countries are in the lead. As a developing country, China is still in its infancy. Most of the theoretical research is empty, the theoretical system of environmental accounting information disclosure has not yet been formed, and there is no specific guideline in actual operation. It is difficult to effectively integrate with practice, and the construction of the theoretical system cannot keep up the pace of economic development. Besides, most of them are general research, there is no targeted specific research and guidance, and environmental accounting standards have not yet been established, resulting in a lack of standardized guidelines in practice. At present, Chinese experts and scholars should focus on how to use foreign research results and successful practical experience to conduct in-depth theoretical research and practical testing in accordance with Chinese national conditions to effectively promote the development of Chinese environmental accounting information disclosure field, thereby contributing to the sustainability of the world economy. Our study makes the following contributions to the literature. First, different from previous studies based on two-party game subjects, this article constructs an evolutionary game model between multiple subjects. On the basis of corporate entities, two external entities, media and investors, have been added to participate in the disclosure of corporate environmental accounting information. Second, this article combines system dynamics to simulate the game process between the three parties, which can effectively analyze the internal game mechanism. Third, as we all know, China is an emerging country and the largest developing country in the world. Therefore, our study selects China as the research object, hoping to provide feasible suggestions for the sustainable development of the economy of developing countries.

## Methodology

### Evolutionary game analysis of corporate environmental accounting information disclosure

#### Evolutionary game theory basis

Traditional game theory emphasizes that the subject of the game is an "entirely rational economic man", that is, the actor has omniscience and omnipotence, and his decision-making plan can be maximized, but this is difficult to achieve in the actual economic environment. However, evolutionary game theory based on "bounded rationality" makes up for this deficiency. In Herbert Simon’s bounded rational decision-making theory, "economic man" is replaced by "social man", and the theory emphasizes that decision-makers pursue "satisfaction" standards in decision-making, rather than optimal ones. In the evolutionary game model, participants have bounded rationality, which means that each stakeholder cannot obtain the optimal strategy through a game, but needs to go through repeated trials and errors, learn and improve their past strategies so as to make behavioral decisions that are more suitable for their current situation. Therefore, the evolutionary game can be used to truly analyze the dynamic game process between enterprises, investors and the media, which can provide scientific and reasonable suggestions for the government and other regulatory agencies to correctly guide enterprises to truly disclose environmental accounting information and actively assume social responsibilities.

#### Evolutionary game model assumptions

*(1) Game subjects*. The three parties in the game are enterprises, investors, and media, all of which are bounded rational economic agents. Among them, enterprises refer to the listed company, and investors refer to the small and medium stockholders, whose shareholding ratio is small and the shareholding is relatively dispersed, so they do not participate in corporate governance. In order to find strategies with higher returns, each subject constantly observes the behavior of others, adjusts its own strategy, breaks the partial balance, conducts evolutionary interaction, and finally achieves relatively stable state.

*(2) Strategy portfolio*. The company’s strategy set is {legal disclosure, false disclosure, non-disclosure}. That is to say, there are two major types of companies’ strategic choices: disclosure and non-disclosure. Disclosure is divided into "legal disclosure" and "false disclosure". "legal disclosure" means that the company provides environmental accounting information legally, compliantly, truthfully and completely in accordance with relevant laws and regulations. "false disclosure" refers to companies providing false environmental accounting information by forging financial data, while "non-disclosure" refers to companies concealing environmental accounting information. The investor’s strategy set is {identification, non-identification}. "identification" means that the investor discovers that the enterprise has false or incomplete disclosure after investing a certain identification cost. The media’s strategy set is {report, non-report}. "report" refers to the media’s public disclosure of corporate environmental accounting information collected through its investigation, including positive reports on the true disclosure of companies, and false disclosure or non-disclosure behavioral negative reports. Under the condition of bounded rationality, it is assumed that the probability that a company chooses a disclosure strategy is *x*, then the probability of non-disclosure is (1 − *x*), and the probability of legal disclosure is *k*, then the probability of false disclosure is 1 − *k*; The probability that the investor recognizes the illegal disclosure behavior of the enterprise is *y*, then the probability that the illegal disclosure behavior of the enterprise is not recognized is (1 − *y*); the probability that the media chooses to report is *z*, the probability that the media does not report is (1 − *z*), and *k*, *x*, *y*, *z* ∈ [0,1]. The strategy combination of the three parties in the game is shown in Table 1.

**Table 1:** Tripartite game strategy combination of enterprise, investor and media.

Game strategy	Investors identify (*y*)		Investors not identify (1 − *y*)	
	Media report (*z*)	Media not report (1 − *z*)	Media report (*z*)	Media not report (1 − *z*)
Company disclose legally *kx*	(A1,B1,C1)	(A2,B2,C2)	(A3,B3,C3)	(A4,B4,C4)
Company disclose falsely (1 − *k*)*x*	(A5,B5,C5)	(A6,B6,C6)	(A7,B7,C7)	(A8,B8,C8)
Company not disclose at all 1 − *x*	(A9,B9,C9)	(A10,B10,C10)	(A11,B11,C11)	(A12,B12,C12)

*(3) Parameter setting*. Suppose the probability that the government regulates the corporate environmental accounting information disclosure is *ρ* and *ρ* ∈ [0,1]. Assuming that all parameters can be measured in currency, the relevant parameters are set as follows:

*α*: The cost of data collection, confirmation, measurement and other procedures incurred by companies for disclosing environmental accounting information;

*β*: The cost of environmental protection investment in order to improve the environmental performance of the report when the company discloses according to the law;

*ε*: The enterprise shall bear the opportunity cost caused by the leakage of commercial secrets due to the disclosure according to the law;

*λ*: When the government supervises, the government rewards that the enterprise receives for disclosure according to law;

*μ*: The comprehensive benefits obtained from the positive media reports on the company’s legal disclosure;

*ν*: When the government supervises, the company will be punished by the government for false disclosure or non-disclosure of environmental accounting information;

*θ*: The media exposed the negative impact of the company’s false disclosure or complete non-disclosure behavior, such as reputation loss and investment reduction;

*ω*: The identification cost paid by investors before making investment decisions;

*ψ*: Uncertainty losses incurred by investors due to their failure to identify the company’s false disclosure or non-disclosure behavior are the unexpected losses. Specifically, the investor loss is *ψ*_11_ when the company falsely discloses and the media reports, *ψ*_12_ when the company falsely discloses but the media does not report, *ψ*_21_ when the company does not disclose but the media reports, and *ψ*_22_ when the company does not disclose but the media does not report;

*η*: Investment income received by investors;

*σ*: The amount of capital invested by the enterprise accepted by investors;

*τ*: The cost of media participation in reporting;

*ξ*: Media gains from reporting. When media reports, print media and electronic media can obtain economic benefits through circulation and clicks respectively;

*κ*: When the government regulates, the government rewards the media for actively participating in reporting, and it is assumed that the government rewards positive and negative media reports at the same amount.

*(4) Income matrix*.

#### Evolutionary game model establishment

(1) According to Tables 1 and 2, it can be seen that the expected return functions of companies adopting the strategies of "disclosure according to law", "false disclosure" and "no disclosure at all" during the game are respectively *U*_*x*1_, *U*_*x*2_ and *U*_*x*3_, and the average return function is ${\bar{U}}_{x}$, then the return functions are:

**Table 2:** Tripartite game strategy combination of enterprise, investor and media.

Strategies	Company	Investors	Media
(A1,B1,C1)	*ρλ* + *μ* + *σ* − *α* − *β* − *ε*	*η* − *ω*	*ξ* + *ρκ* − *τ*
(A2,B2,C2)	*ρλ* + *σ* − *α* − *β* − *ε*	*η* − *ω*	0
(A3,B3,C3)	*ρλ* + *μ* + *σ* − *α* − *β* − *ε*	*η* − *ω*	*ξ* + *ρκ* − *τ*
(A4,B4,C4)	*ρλ* + *σ* − *α* − *β* − *ε*	*η* − *ω*	0
(A5,B5,C5)	−*ρν* − *θ* − *α*	−*ω*	*ξ* + *ρκ* − *τ*
(A6,B6,C6)	−*ρν* − *α*	−*ω*	0
(A7,B7,C7)	*σ* − *ρν* − *θ* − *α*	*η* − *ω* − *ψ*_11_	*ξ* + *ρκ* − *τ*
(A8,B8,C8)	*σ* − *ρν* − *α*	*η* − *ω* − *ψ*_12_	0
(A9,B9,C9)	−*ρν* − *θ*	−*ω*	*ξ* + *ρκ* − *τ*
(A10,B10,C10)	−*ρν*	−*ω*	0
(A11,B11,C11)	*σ* − *ρν* − *θ*	*η* − *ω* − *ψ*_21_	*ξ* + *ρκ* − *τ*
(A12,B12,C12)	*σ* − *ρν*	*η* − *ω* − *ψ*_22_	0


$$\begin{array}{ll} U_{x1} & =\left(\rho \lambda +\mu +\sigma -\alpha -\beta -\varepsilon \right)yz+\left(\rho \lambda +\sigma -\alpha -\beta -\varepsilon \right)y\left(1-z\right)+\\ & \;\left(\rho \lambda +\mu +\sigma -\alpha -\beta -\varepsilon \right)\left(1-y\right)z+\left(\rho \lambda +\sigma -\alpha -\beta -\varepsilon \right)\left(1-y\right)\left(1-z\right)\\ & =\mu z+\rho \lambda +\sigma -\alpha -\beta -\varepsilon \end{array}$$ (1)

$$\begin{array}{ll} U_{x2} & =\left(-\rho \nu -\theta -\alpha \right)yz+\left(-\rho \nu -\alpha \right)y\left(1-z\right)+\left(\sigma -\rho \nu -\theta -\alpha \right)\left(1-y\right)z+\left(\sigma -\rho \nu -\alpha \right)\left(1-y\right)\left(1-z\right)\\ & =\sigma \left(1-y\right)-\theta z-\rho \nu -\alpha \end{array}$$ (2)

$$\begin{array}{ll} U_{x3} & =\left(-\rho \nu -\theta \right)yz+\left(-\rho \nu \right)y\left(1-z\right)+\left(\sigma -\rho \nu -\theta \right)\left(1-y\right)z+\left(\sigma -\rho \nu \right)\left(1-y\right)\left(1-z\right)\\ & =\sigma \left(1-y\right)-\theta z-\rho \nu \end{array}$$ (3)

$$\begin{array}{ll} {\bar{U}}_{x} & =kxU_{x1}+\left(1-k\right)xU_{x2}+\left(1-x\right)U_{x3}\\ & =kx\left(\mu z+\rho \lambda +\sigma -\alpha -\beta -\varepsilon \right)+\left(1-kx\right)\left[\sigma \left(1-y\right)-\theta z-\rho v\right]+\alpha \left(k-1\right)x \end{array}$$ (4)

Then the replicated dynamic equation for enterprises to adopt the strategy of "disclosure according to law" is:

$$\begin{array}{ll} F\left(kx\right) & =\frac{d\left(kx\right)}{dt}=kx\left(U_{x1}-{\bar{U}}_{x}\right)\\ & =kx\left\{\left(1-kx\right)\left(\mu z+\rho \lambda +\sigma -\alpha -\beta -\varepsilon \right)+\left(kx-1\right)\left[\sigma \left(1-y\right)-\theta z-\rho \nu -\alpha \right]-\alpha \left(1-x\right)\right\} \end{array}$$ (5)

(2) The expected return functions of the "identified" and "unidentified" strategies used by investors in the game are *U*_*y*1_ and *U*_*y*2_, and the average return function is ${\bar{U}}_{y}$, then the return functions are:

$$\begin{array}{ll} U_{y1} & =\left(\eta -\omega \right)kxz+\left(\eta -\omega \right)kx\left(1-z\right)+\left(-\omega \right)\left(1-k\right)xz+\left(-\omega \right)\left(1-k\right)x\left(1-z\right)+\left(-\omega \right)\left(1-x\right)z\\ & +\left(-\omega \right)\left(1-x\right)\left(1-z\right)\\ & =\eta kx-\omega \end{array}$$ (6)

Uy2=η−ωkxz+η−ωkx1−z+η−ω−ψ111−kxz+η−ω−ψ121−kx1−z+η−ω−ψ211−xz+η−ω−ψ221−x1−z=η−ω+k−1xzψ11−ψ12+ψ12+x−1zψ21−ψ22+ψ22 (7)

$$\begin{array}{ll} {\bar{U}}_{y} & =yU_{y1}+\left(1-y\right)U_{y2}\\ & =\eta \kappa xy-\eta y+\eta -\omega +\left(1-y\right)\left\{\left(k-1\right)x\left[z\left(\psi_{11}-\psi_{12}+\psi_{12}\right)\right]+\left(x-1\right)\left[z\left(\psi_{21}-\psi_{22}\right)+\psi_{22}\right]\right\} \end{array}$$ (8)

Then the replicated dynamic equation for investors to adopt the strategy of "identified" is:

$$\begin{array}{ll} F\left(y\right) & =\frac{dy}{dt}=y\left(U_{y1}-{\bar{U}}_{y}\right)\\ & =y\left(1-y\right)\left\{\eta \left(kx-1\right)+\left(1-k\right)x\left[z\left(\psi_{11}-\psi_{12}\right)+\psi_{12}\right]+\left(1-x\right)\left[z\left(\psi_{21}-\psi_{22}\right)+\psi_{22}\right]\right\} \end{array}$$ (9)

(3) The expected return functions of the “report” and “non-report” strategies adopted by the media in the game are respectively *U*_*z*1_ and *U*_*z*2_, and the average return function is ${\bar{U}}_{z}$, then the return functions are:

$$\begin{array}{ll} U_{z1} & =\left(\xi +\rho \kappa -\tau \right)kxy+\left(\xi +\rho \kappa -\tau \right)kx\left(1-y\right)+\left(\xi +\rho \kappa -\tau \right)\left(1-k\right)xy\\ & +\left(\xi +\rho \kappa -\tau \right)\left(1-k\right)x\left(1-y\right)+\left(\xi +\rho \kappa -\tau \right)\left(1-x\right)y+\left(\xi +\rho \kappa -\tau \right)\left(1-x\right)\left(1-y\right)\\ & =\xi +\rho \kappa -\tau \end{array}$$ (10)

$$U_{z2}=0$$ (11)

$${\bar{U}}_{z}=zU_{z1}+\left(1-z\right)U_{z2}=\left(\xi +\rho \kappa -\tau \right)z$$ (12)

Then the replicated dynamic equation for media to adopt the strategy of “report” is:

$$F\left(z\right)=\frac{dz}{dt}=z\left(U_{z1}-{\overset{-}{U}}_{z}\right)=\left(\xi +\rho \kappa -\tau \right)z\left(1-z\right)$$ (13)

Combining formula *F*(*kx*), formula *F*(*y*) and formula *F*(*z*) together can obtain the dynamic equations of duplication of enterprises, investors and media, namely

$$\left\{\begin{array}{l} F\left(kx\right)=kx\left\{\left(1-kx\right)\left(\mu z+\rho \lambda +\sigma -\alpha -\beta -\varepsilon \right)+\left(kx-1\right)\left[\sigma \left(1-y\right)-\theta z-\rho \nu -\alpha \right]-\alpha \left(1-x\right)\right\}\\ F\left(y\right)=y\left(1-y\right)\left\{\eta \left(kx-1\right)+\left(1-k\right)x\left[z\left(\psi_{11}-\psi_{12}\right)+\psi_{12}\right]+\left(1-x\right)\left[z\left(\psi_{21}-\psi_{22}\right)+\psi_{22}\right]\right\}\\ F\left(z\right)=\left(\xi +\rho \kappa -\tau \right)z\left(1-z\right) \end{array}\right.$$ (14)

#### Stability analysis of equilibrium

Let the 3 replication dynamic equations be equal to 0, and 12 local equilibrium points can be obtained, as shown in Table 3.

**Table 3:** The value of the equilibrium point.

Equilibrium strategy	Value of k	Value of x	Value of y	Value of z
E1(legal disclosure, identification, report)	1	1	1	1
E2(legal disclosure, identification, non-report)	1	1	1	0
E3(legal disclosure, non-identification, report)	1	1	0	1
E4(legal disclosure, non-identification, non-report)	1	1	0	0
E5(false disclosure, identification, report)	0	1	1	1
E6(false disclosure, identification, non-report)	0	1	1	0
E7(false disclosure, non-identification, report)	0	1	0	1
E8(false disclosure, non-identification, non-report)	0	1	0	0
E9(non-disclosure, identification, report)	-	0	1	1
E10(non-disclosure, identification, non-report)	-	0	1	0
E11(non-disclosure, non-identification, report)	-	0	0	1
E12(non-disclosure, non-identification, non-report)	-	0	0	0

Jacobian matrix is:

$$J=\left[\begin{array}{cccc} J_{11} & J_{12} & J_{13} & J_{14}\\ J_{21} & J_{22} & J_{23} & J_{24}\\ J_{31} & J_{32} & J_{33} & J_{34} \end{array}\right]=\left[\begin{array}{cccc} \frac{\partial F\left(kx\right)}{\partial k} & \frac{\partial F\left(kx\right)}{\partial x} & \frac{\partial F\left(kx\right)}{\partial y} & \frac{\partial F\left(kx\right)}{\partial z}\\ \frac{\partial F\left(y\right)}{\partial k} & \frac{\partial F\left(y\right)}{\partial x} & \frac{\partial F\left(y\right)}{\partial y} & \frac{\partial F\left(y\right)}{\partial z}\\ \frac{\partial F\left(z\right)}{\partial k} & \frac{\partial F\left(z\right)}{\partial x} & \frac{\partial F\left(z\right)}{\partial y} & \frac{\partial F\left(z\right)}{\partial z} \end{array}\right]$$ (15)

Among them,

$$\begin{array}{l} J_{\text{11}}=\left(x-2kx^{2}\right)\left(\mu z+\rho \lambda +\sigma -\alpha -\beta -\varepsilon \right)+\left(2kx^{2}-x\right)\left[\sigma \left(1-y\right)-\theta z-\rho \nu -\alpha \right]-\alpha x\left(1-x\right);\\ J_{\text{12}}=\left(k-2k^{2}x\right)\left(\mu z+\rho \lambda +\sigma -\alpha -\beta -\varepsilon \right)+\left(2k^{2}x-k\right)\left[\sigma \left(1-y\right)-\theta z-\rho \nu -\alpha \right]-\alpha k\left(1-2x\right)J_{\text{13}}=\sigma kx\left(1-kx\right);\\ J_{\text{14}}=kx\left(1-kx\right)\left(\mu +\theta \right);J_{\text{21}}=xy\left(1-y\right)\left[\eta -z\left(\psi_{11}-\psi_{12}\right)-\psi_{12}\right];\\ J_{\text{22}}=y\left(1-y\right)\left\{\eta k+\left(1-k\right)\left[z\left(\psi_{11}-\psi_{12}\right)+\psi_{12}\right]-\left[z\left(\psi_{21}-\psi_{22}\right)+\psi_{22}\right]\right\};\\ J_{\text{23}}=\left(\text{1}-\text{2y}\right)\left\{\eta \left(kx-1\right)+\left(1-k\right)x\left[z\left(\psi_{11}-\psi_{12}\right)+\psi_{12}\right]+\left(1-x\right)\left[z\left(\psi_{21}-\psi_{22}\right)+\psi_{22}\right]\right\};\\ J_{24}=y\left(1-y\right)\left[\left(1-k\right)x\left(\psi_{11}-\psi_{12}\right)+\left(1-x\right)\left(\psi_{21}-\psi_{22}\right)\right]J_{\text{31}}=\text{0};J_{32}=\text{0};J_{\text{33}}=0;J_{\text{34}}=\left(\xi +\rho \kappa -\tau \right)\left(1-2z\right). \end{array}$$

From the unequal number of rows and columns of the Jacobian matrix, it can be seen that it is not a square matrix, which cannot determine the stability of the remaining equilibrium points, so it cannot indicate that the equilibrium point that makes the three-party game reach a stable point must exist. This requires further exploration in conjunction with the system dynamics simulation model.

### System dynamics simulation research

**Fig 1:**
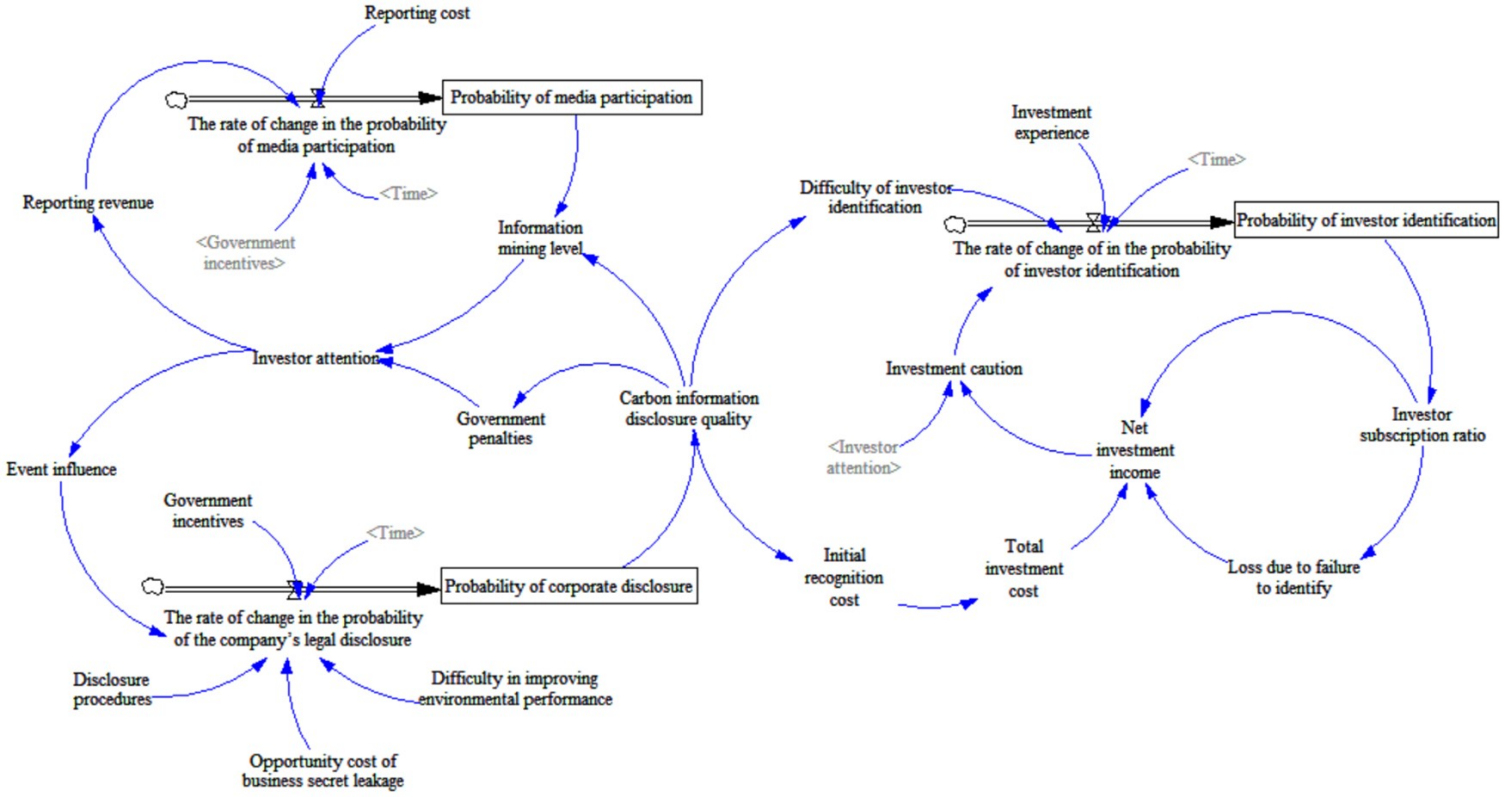
SD simulation model for enterprises, investors and media.

According to the above analysis, Vensim PLE software is used to establish the SD model of the three main entities of enterprises, investors and media in environmental accounting information disclosure, as shown in Fig 1.

The SD model is mainly composed of 3 state variables, 3 rate variables, 6 constants and 13 auxiliary variables, with a total of 25 variables. This research uses expert scoring method and questionnaire survey method to assign values to variables and constants based on the actual situation in China. Fortunately, many professors, associate professors and doctoral students from Nanjing University, Jiangsu University and Yangzhou University were invited to score each factor on a scale of 0 to 1, and the final score is formed according to the corresponding weight. The initial values of some variables are surveyed by netizen, and the corresponding scores are obtained by weighting after the effective questionnaires are collected. For the convenience of simulation analysis, the results are kept to one decimal place, as shown in the Tables 4 and 5.

**Table 4:** The constants of SD model.

Variable names	Value
The degree of cumbersome disclosure procedures	0.6
Proportion of opportunity cost of business secret leakage	0.5
Difficulty in improving environmental performance	0.9
Government incentives	0.3
Investment experience level	0.1
Reporting cost ratio	0.6

**Table 5:** The initial values of SD model.

Variable names	Value
Probability of corporate disclosure	0.3
Probability of Investor identification	0.3
Probability of media coverage	0.4

The flow diagram is the overall reflection of the system structure, and the equation describes the quantitative relationship between the elements in the structure. The equations among the main variables in the SD model are shown in Table 6.

**Table 6:** The equations between main variables.

Variable types	Variable names	Expression and initial assignment
Level variable	Probability of corporate disclosure	= INTEG(The rate of change in the probability of the company’s legal disclosure, 0.3)
	Probability of investor identification	= INTEG(The rate of change of in the probability of investor identification, 0.3)
	Probability of media participation	= INTEG(The rate of change in the probability of media participation, 0.4)
Rate variable	The rate of change in the probability of the company’s legal disclosure	= (Event influence*0.2+Government incentives*0.3-Disclosure procedures*0.2-Opportunity cost of business secret leakage*0.1-Difficulty in improving environmental performance*0.2)/Time
	The rate of change of in the probability of investor identification	= (Investment experience*0.4 + Investment caution*0.1- Difficulty of investor identification*0.5)/Time
	The rate of change in the probability of media participation	= (Reporting revenue*0.2 + Government incentives*0.4- Reporting cost*0.4)/Time
Auxiliary variable	Environmental accounting information disclosure quality	= IF THEN ELSE(0< = Probability of corporate disclosure: AND: Probability of corporate disclosure< = 0.5, 1/3*SIN(10*Probability of corporate disclosure) + 1/2, Probability of corporate disclosure)
	Government penalties	= EXP(-environmental accounting information disclosure quality)
	Investor attention	= Government penalties*0.4+Information mining level*0.6
	Event influence	= Investor attention*1.7
	Investor subscription ratio	= 2/3*EXP(-Probability of investor identification/3)
	Loss due to failure to identify	= EXP(Investor subscription ratio)
	Net investment income	= Investor subscription ratio*0.3—Total investment cost*0.2—Loss due to failure to identify*0.5
	Investment caution	= Investor attention*0.64-Net investment income*0.36
	Initial recognition cost	= IF THEN ELSE(0< = environmental accounting information disclosure quality:AND:environmental accounting information disclosure quality< = 1, 1/(environmental accounting information disclosure quality+1)-1/3, 0.1)
	Total investment cost	= Initial recognition cost*1.2
	Difficulty of investor identification	= IF THEN ELSE(0< = environmental accounting information disclosure quality:AND:environmental accounting information disclosure quality< = 0.5, 1/4*environmental accounting information disclosure quality^2–2*environmental accounting information disclosure quality+1, environmental accounting information disclosure quality^2-environmental accounting information disclosure quality+0.3125)
	Information mining level	= Probability of media participation*0.5-environmental accounting information disclosure quality*0.5+0.9
	Reporting revenue	= Investor attention*0.5

## Simulation results and discussion

### Initial simulation analysis

Based on the above SD model, the initial value is simulated first to analyze the initial operating results between the three parties. Use Vensim software to set INITIAL TIME = 1, FINAL TIME = 50, TIME STEP = 1, Units for time: Day. In the simulation diagram, the horizontal axis represents time, and the vertical axis represents the probability of different agents’ strategy choices.

**Fig 2:**
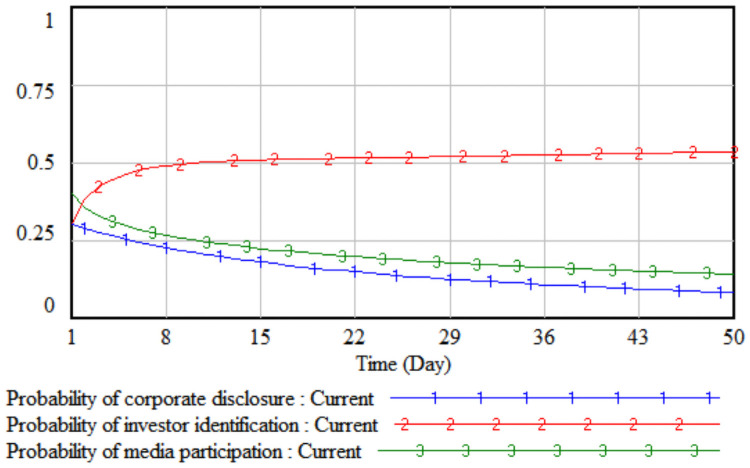
The initial simulation results of the three parties.

It can be seen from Fig 2 that when the model runs at the initial value, the media report probability will show a downward trend with the passage of time. When it reaches 0.14, it will gradually stabilize; the probability of companies choosing to disclose according to law and media report both show a slow downward trend. Eventually it gradually tends to 0.08 on the 48th day; investors gradually accumulate investment experience over time, and the identification probability shows an upward trend, and finally reaches an equilibrium state of 0.54 on the 50th day.

### Simulation analysis of external variables

In order to further study the impact of external variables on the SD model, this article selects four external variables from the perspective of multi-agent, including government incentives, disclosure procedures, difficulty in improving environmental performance and opportunity cost of business secret leakage. Two external variables, namely, the investment experience level and reporting cost ratio, are selected from investors and media subjects respectively, and a comprehensive strategic analysis is finally carried out around these six external variables. Since government incentives and investment experience levels are positive influencing factors, the parameter values of these two variables are increased in proportion to the initial values to analyze the choice of strategies among entities. The other four external variables such as the cumbersomeness of the disclosure procedure are all negative factors, so the parameter values of these four variables are reduced in equal proportions based on the initial values.

#### Analysis of external variables in enterprise main body

**Fig 3:**
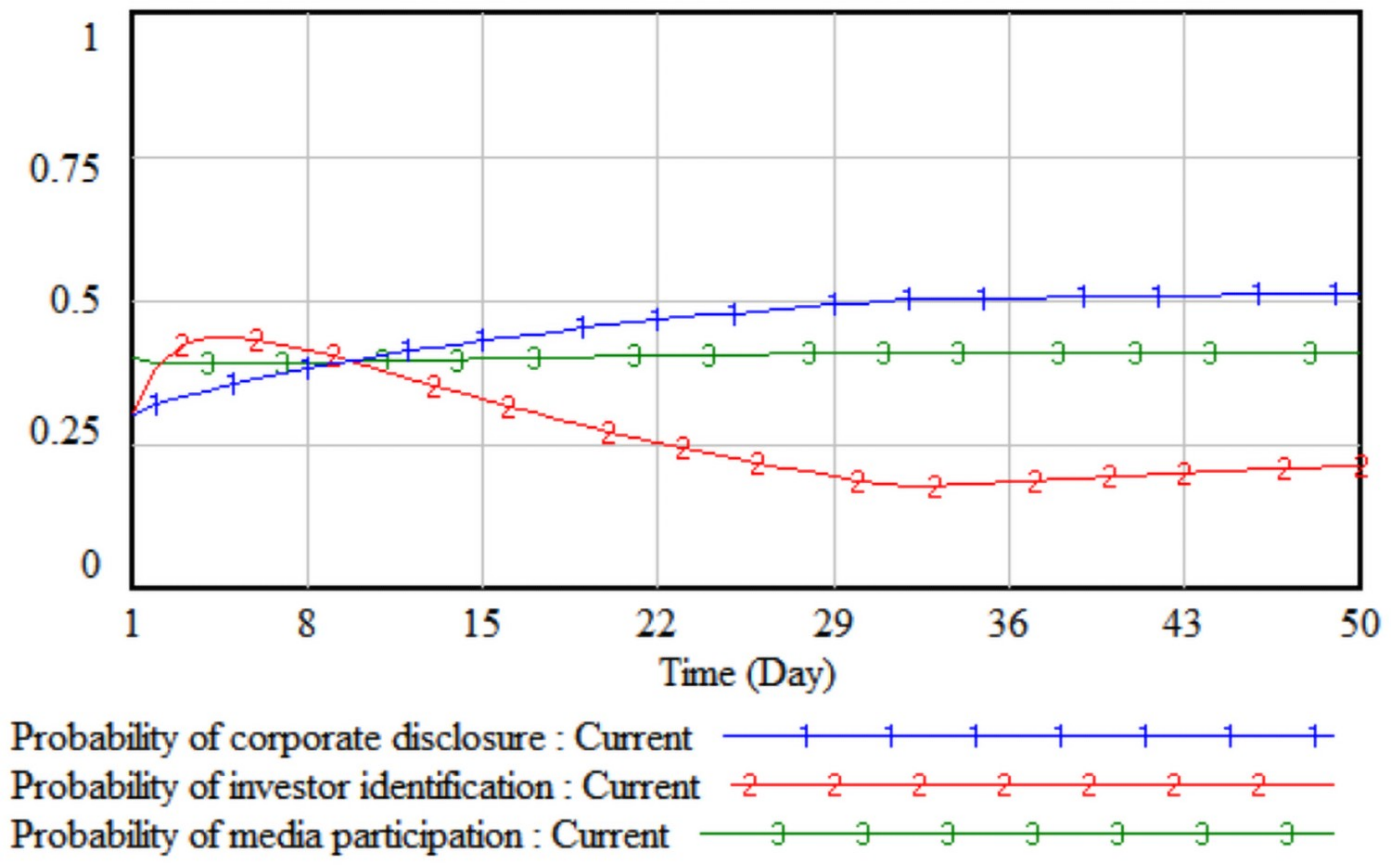
When government incentives are 0.4.

**Fig 4:**
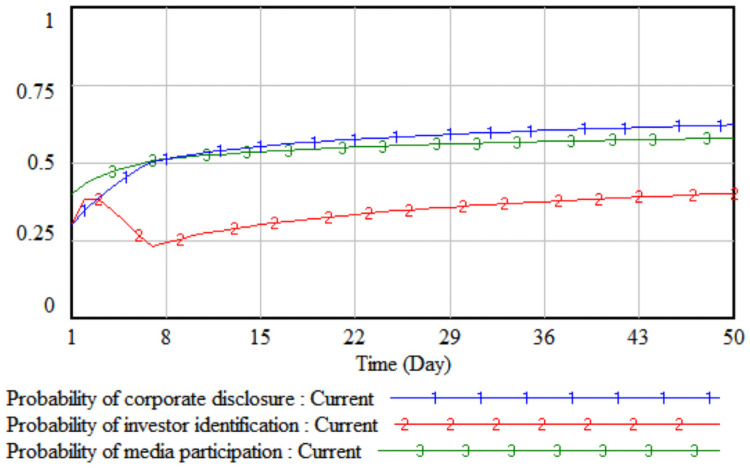
When government incentives are 0.5.

*1*. *Government incentives*. By changing the intensity of government incentives, it simulates the impact of different incentive levels adopted by the government on the strategic choices of the tripartite entities. The government incentives are increased by 10% and 20% respectively on the basis of the initial value, that is, set to 0.4 and 0.5 respectively. The three-party evolutionary game results corresponding to the two simulations are shown in Figs 3 and 4.

It can be seen from Figs 3 and 4 that when the government’s incentives are increased by 10%, the media report probability stabilizes at the initial value of 0.41, that is, the media is in a wait-and-see situation under the comprehensive trade-off of the cost-benefit principle. At this time, the media is still more likely to adopt non-reporting behaviors; when the government’s incentives increase by 20%, the rate of increase in the probability of media report will increase significantly. Comparing Figs 3 and 4, it can be seen that when the incentives are the same, under the stimulus of increased corporate sensitivity, a smaller government incentive can change the direction of the company’s legal disclosure strategy, and the growth rate of the company’s disclosure according to law in probability is always slightly higher than that of the media. When the lawful disclosure probability of enterprises in the whole society is generally high, the quality of environmental accounting information disclosure will be significantly improved. At this time, the difficulty of investor identification and the initial identification cost will gradually decrease, so that the probability of investor identification will increase over time. The obvious growth will eventually promote the evolution of the strategic combination (legal disclosure, identification, and report) of the three parties, including companies, investors, and the media.

**Fig 5:**
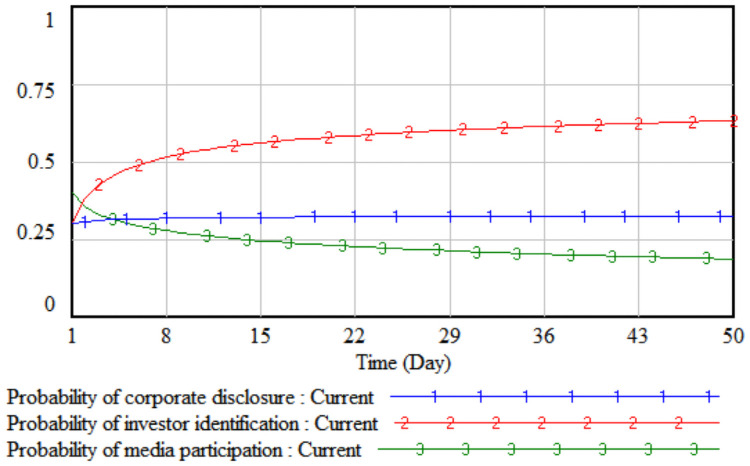
When the degree is 0.5.

**Fig 6:**
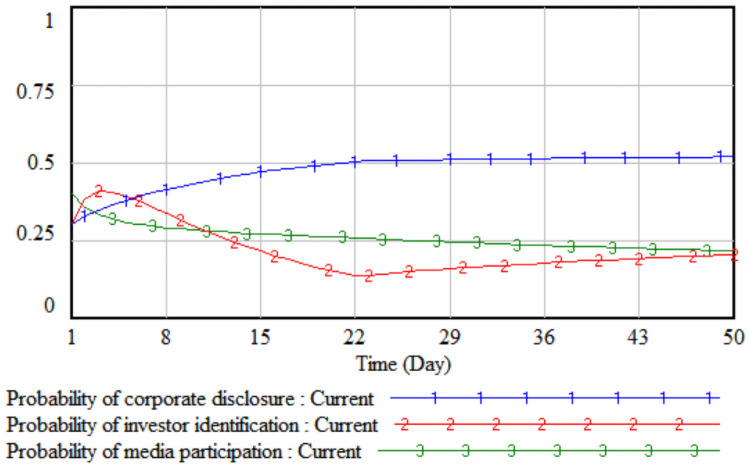
When the degree is 0.4.

*2*. *The degree of cumbersome disclosure procedures*. By changing the value of the cumbersome degree of the disclosure procedure, the impact of the disclosure procedure on the strategic choices of the three parties is simulated. The cumbersomeness of the disclosure procedure is reduced by 10% and 20% respectively on the basis of the initial value, that is, set to 0.5 and 0.4, respectively, and the three-party evolutionary game results corresponding to the two simulations are shown in Figs 5 and 6.

It can be seen from Figs 5 and 6 that when the cumbersomeness of the disclosure procedure is reduced by 10%, the increase in the probability of a company choosing to disclose according to law is not obvious, and it stabilizes when the probability reaches 0.32. At this time, the company is still more likely not to disclose environmental accounting information according to law. When the cumbersomeness of the disclosure procedure is reduced by 20%, the rate of increase in the probability of a company’s legal disclosure is significantly higher than that shown in Fig 5. The evolution path of investors has shown an "S"-shaped change over time. Initially, its growth rate was relatively fast. However, because the probability of corporate disclosure according to law is still low, and the quality of environmental information disclosure is poor, the difficulty of identifying investors is short. It is difficult to decrease rapidly within a period of time. An inflection point appeared on the third day, and then the growth rate gradually slowed down. It can be seen that the method of blindly simplifying the disclosure procedures to prompt companies to disclose according to law has little effect in the short term, and it is difficult to see immediate results. The tripartite strategic combination will eventually evolve in the direction of (non-disclosure according to law, non-identification, non-report).

**Fig 7:**
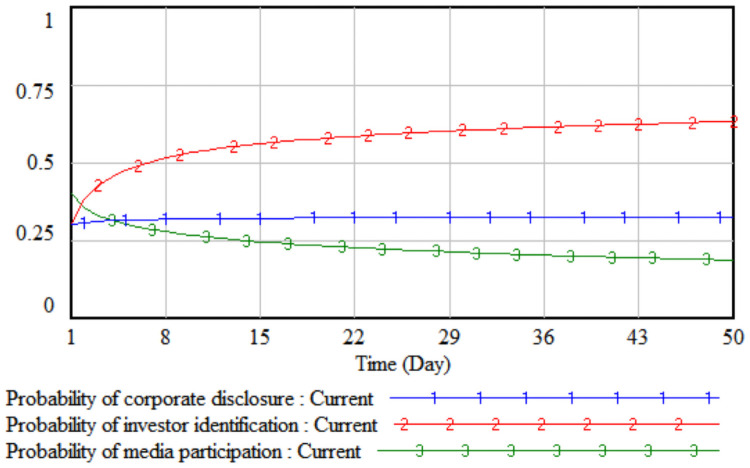
When the difficulty is 0.8.

**Fig 8:**
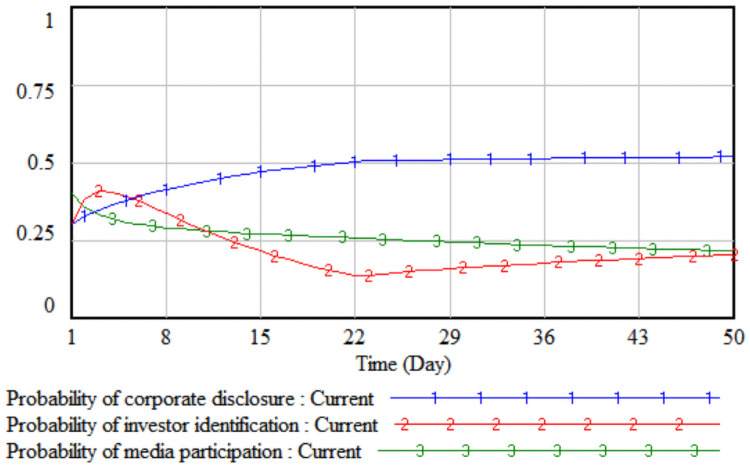
When the difficulty is 0.7.

*3*. *Difficulty in improving environmental performance*. By changing the value of the difficulty of environmental performance improvement, the impact of the difficulty of improving environmental performance on the strategic choices of the three parties is simulated. The difficulty of improving the environmental performance is reduced by 10% and 20% respectively on the basis of the initial value, that is, set to 0.8 and 0.7 respectively, then the three-party evolutionary game results corresponding to the two simulations are shown in Figs 7 and 8.

It can be seen from Figs 7 and 8 that when the difficulty in improving environmental performance is reduced by 10%, the probability that a company chooses to disclose according to law is basically stable at the initial value of 0.32; when it is reduced by 20%, as the difficulty of improving environmental performance has fallen sharply, the cost of legal disclosure by enterprises is much less than the comprehensive income, which has realized the real burden reduction of enterprises according to legal disclosure, so the probability of the company’s legal disclosure has continued to increase. As the probability of a company’s legal disclosure increases, the cost of investor identification will continue to decline, and the probability of investor identification will gradually increase after a short period of fluctuation. Comparing Figs 5–8, when the cumbersomeness of the disclosure procedure is reduced to 0.4 and the difficulty of environmental performance improvement is reduced to 0.7, it has the same effect. From a long-term perspective, only by continuously reducing the difficulty of improving environmental performance will the three parties, including companies, investors, and the media, evolve the strategic direction (legal disclosure, identification, non-report).

**Fig 9:**
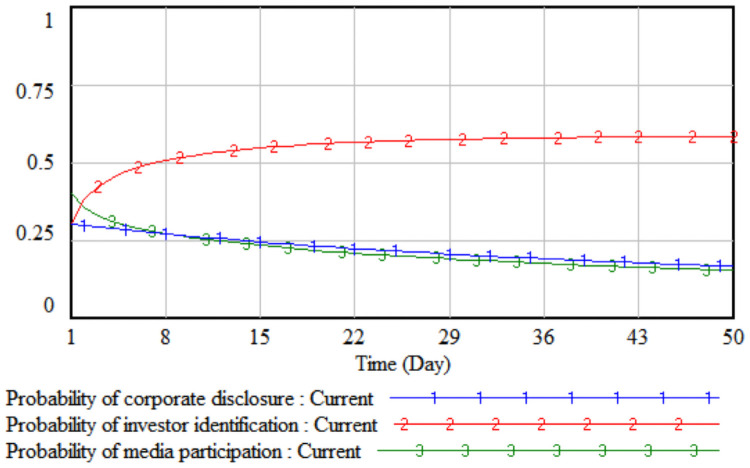
When the proportion is 0.4.

**Fig 10:**
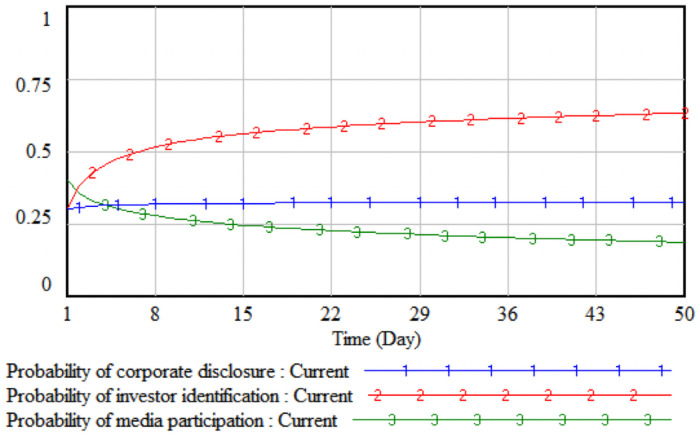
When the proportion is 0.3.

*4*. *Proportion of opportunity cost of commercial secret leakage*. By changing the proportion of the opportunity cost of the leakage of trade secrets, the impact of the opportunity cost of the leakage of trade secrets on the strategic choices of the three parties is simulated. The opportunity cost ratio is reduced by 10% and 20% respectively on the basis of the initial value that is set to 0.4 and 0.3 respectively, then the three-party evolutionary game results corresponding to the two simulations are shown in Figs 9 and 10.

It can be seen from Figs 9 and 10 that when the proportion of the opportunity cost of business secret leakage is reduced by 10%, the company’s legal disclosure probability will not quickly converge to 0, but still shows a downward trend; when the proportion of the opportunity cost of the leakage of business secrets is reduced by 20%, the probability of legal disclosure of enterprises has increased slightly in the first 5 days, but the growth rate has been slow, and has stabilized at 0.32 since the sixth day. The reason is that the opportunity cost of business secret leakage accounts for a relatively small proportion of the company’s decision to disclose behaviors in accordance with the law, while the cost of procedures and environmental performance improvement costs are relatively large. Only when the opportunity cost of business secret leakage is extremely low will reverse the strategy, but this is not in line with the reality, so it is difficult to promote the company’s strategic transformation by strengthening the protection of business secrets. In addition, the probability of media and investors has not changed much from the initial value. It can be concluded that the opportunity cost of the leakage of business secrets is not the main factor that affects the company’s choice of legally disclosing environmental accounting information strategies. The tripartite strategy portfolio will eventually evolve in the direction of (non-disclosure, identification, non-report).

#### Analysis of external variables in the main body of investors

**Fig 11:**
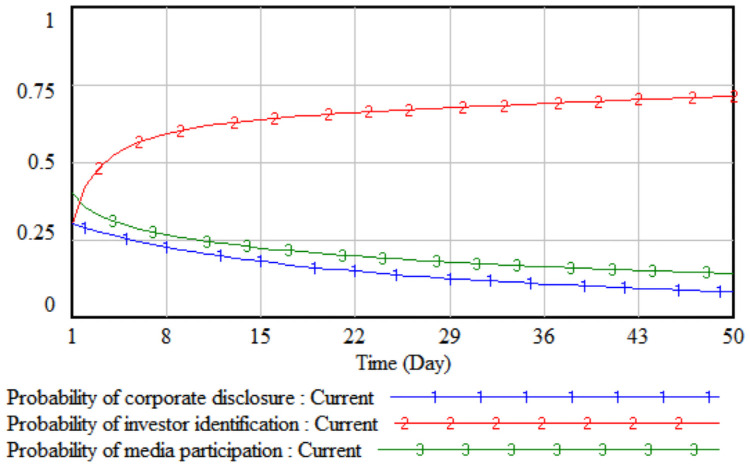
When the level is 0.2.

**Fig 12:**
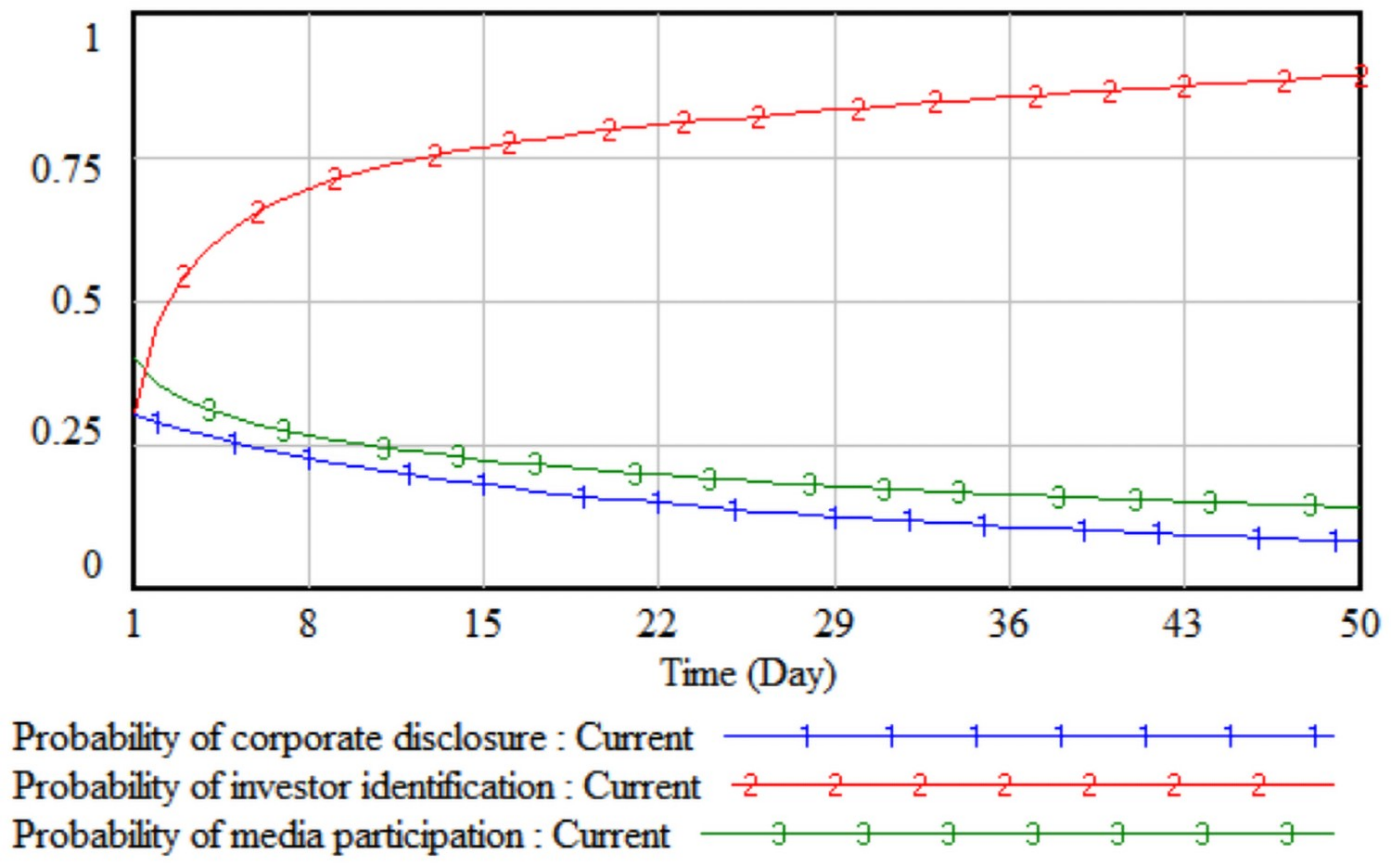
When the level is 0.3.

By changing the level of investment experience, it simulates the impact of the level of investment experience on the strategic choices of the tripartite entities. Increase the experience level by 10% and 20% respectively on the basis of the initial value that is set to 0.2 and 0.3 respectively, and the three-party evolutionary game results corresponding to the two simulations are shown in Figs 11 and 12.

It can be seen from Figs 11 and 12 that when the level of investment experience increases by 10%, investors will sum up the gains and losses based on past investment experience, learn lessons from failed investment projects, and gradually accumulate investment experience, thereby increasing the probability of investor identification, finally stabilized at 0.71 on the 46th day; when the investor protection mechanism is improved by 20%, the investor identification probability continues to increase at a faster rate of change, and finally approaches 0.9. It can be seen that the higher the level of investment experience, the more likely it is to increase the probability of investor identification, but the impact on the choice of corporate and media strategies is not significant, and ultimately makes the tripartite subject of companies, investors and media to (non-disclosure, identification, non-report) evolution of strategic direction.

#### Analysis of external variables in the main body of the media

**Fig 13:**
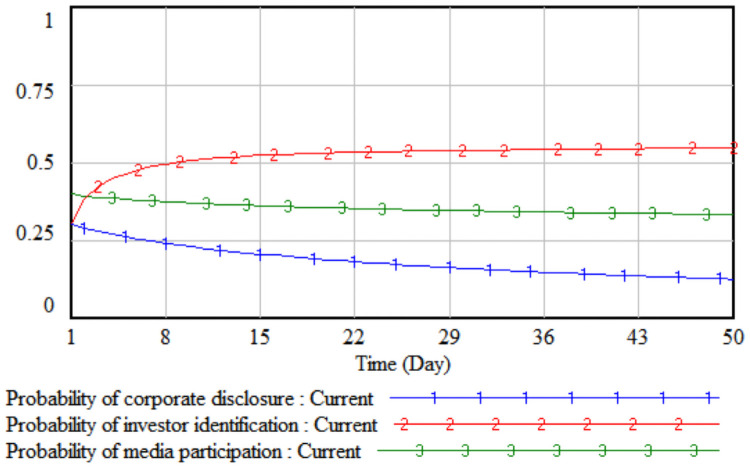
When the cost is 0.5.

**Fig 14:**
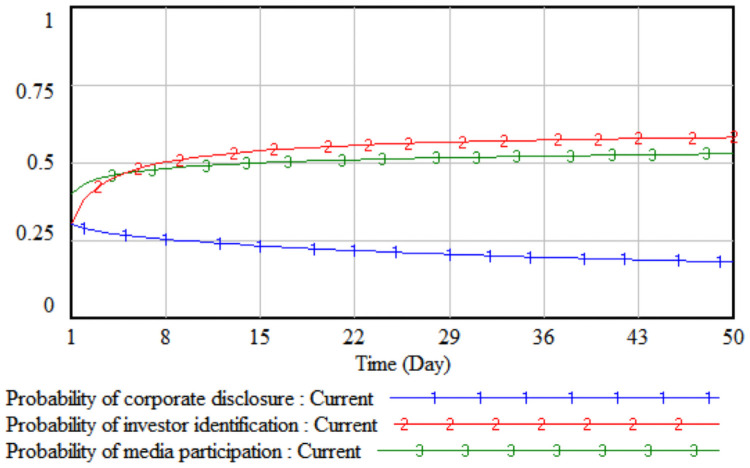
When the cost is 0.4.

By changing the proportion of media reporting costs, it simulates the impact of media reporting costs on the strategic choices of the three parties. If the media report cost ratio is reduced by 10% and 20% respectively on the basis of the initial value, that is, set to 0.5 and 0.4 respectively, the three-party evolutionary game results corresponding to the two simulations are shown in Figs 13 and 14.

It can be seen from Figs 13 and 14 that when the proportion of media report costs is reduced by 10%, the probability of media report still shows a downward trend, but the rate of change is less than the initial simulation result. At this time, with the increase of media exposure, the amount of information obtained by investors has continued to increase, investment experience has become richer, and the probability of identifying information has also increased; when the proportion of media report costs is reduced by 20%, the rate of increase in the probability of media choosing to participate in the report is significantly increased, while the rate of investor identification gradually slowed after reaching 0.50 on the 8th day. The reason is that the media’s mining of corporate environmental accounting information disclosure has reached a certain level, and investors’ attention to relevant information has declined. However, at the same time, the probability of companies choosing to disclose according to law is still on a downward trend, that is, companies will not passively choose to disclose environmental accounting information in accordance with the law as media exposure increases, and most companies are still willing to bear the possible loss of reputation after media exposure and do not actively choose a legal disclosure strategy. When the cost of media reports is reduced to a certain level, the tripartite strategy portfolio will evolve in the direction of (non-disclosure, identification, and report).

## Conclusions

### Summary of research results

This paper takes China as the research object, explores the strategic combination of the three parties by constructing the evolutionary game model of enterprises, investors and media, and uses system dynamics to conduct dynamic simulation analysis of the game process. From the analysis of the model, it can be concluded that for developing countries, the government should appropriately adopt positive incentives to prompt the media to accelerate the conversion strategy, so as to promote the media to actively participate in the supervision of corporate environmental accounting information disclosure. Due to the high sensitivity of enterprises, the government can appropriately reduce the incentives for enterprises to achieve the dual goal of reducing government input costs and pressure on enterprises by the media, and ultimately prompt enterprises to quickly change their strategies. In addition, with the increase in the probability of corporate disclosure by law, the probability of investor identification continues to increase, resulting in the three-party strategy of companies, investors, and the media eventually evolving toward the optimal equilibrium state of (legal disclosure, identification, and report) to promote environmental accounting information disclosure mechanism to work well.

### Managerial insights

From the research of this paper, the efficient environmental accounting information disclosure of enterprises requires the cooperation of multiple parties. However, under the constraints of political environment, economic development, ecological protection and other factors, generally speaking, according to the current situation, due to the late start of environmental information disclosure in China and other developing countries in the world, their practical explorations are still at the initial stage and they generally lack mature and complete theories as guidance. Therefore, they need to learn from the excellent experience of the United States and other developed countries, and at the same time strengthen the government and other external entities’ supervision of enterprises, increase the enthusiasm of enterprises to disclose environmental information, and establish a more complete environmental accounting information disclosure system. In order to establish an effective environmental accounting information disclosure mechanism, the following managerial insights are made for the three main bodies of enterprises, investors and media:

**Enterprises**: The strategic choice of a company is greatly influenced by the value of three variables: the government’s incentives in the main body of the company, the cumbersome degree of disclosure procedures, and the difficulty of improving environmental performance. The greater the government’s incentives, the faster the company’s strategy will change. Therefore, the government should appropriately adopt positive incentives, such as tax cuts, fee reductions, and financial appropriations, to promote corporate conversion strategies, and enable companies to evolve in the direction of legal disclosure strategies. In addition, reducing the cumbersomeness of the disclosure procedure can reduce the burden of the company’s disclosure according to law to a certain extent, but it cannot play a role in curing the root cause. Therefore, while tackling the symptoms, it is necessary to tackle the root cause and fundamentally reduce the difficulty of improving environmental performance, so that the comprehensive income that the company can obtain after legal disclosure is much greater than the disclosure cost, and it can effectively promote the company to adopt legal disclosure. This requires the government to make great efforts. Develop a circular economy, reduce the cost of green production for enterprises in the whole society, while further simplifying the disclosure procedures, and finally clear the obstacles for enterprises to disclose environmental accounting information in accordance with the law.

**Investors**: The simulation found that the identification probability of investors is largely affected by the value of the external variable, the level of investment experience. The higher the level of investment experience is, the greater the probability of investor recognition will be. Therefore, investors should improve their own investment risk awareness, and before making investment decisions, they must consider investment returns and fully weigh investment risks. At the same time, investors should also strengthen their awareness of self-protection, expand the collection channels of investment information through national official websites and mainstream media, and seek legal protection by relying on relevant investor protection systems to reduce losses due to unidentified illegal information to achieve the goal of reducing investment risk.

**Media**: The simulation found that reducing the cost of media reports will attract the media to participate in the supervision of corporate disclosure behavior to a certain extent, and the greater the government’s incentives, the more likely it is to promote the media’s choice of participating in reporting strategies. Therefore, the media should actively participate in the supervision of corporate environmental accounting information disclosure, actively convey the latest government policy guidelines, play the role of external monitors, and promote the effective operation of the environmental accounting information disclosure mechanism. For companies that disclose environmental accounting information in accordance with laws and regulations, the media should increase publicity, create a responsible benchmark corporate image, and form a good atmosphere of lawful disclosure of environmental accounting information in the whole society; for negative companies that have not disclosed environmental accounting information in accordance with the law, the media should seek truth from facts, maintain the independence of news reports, provide investors with timely and true information in the form of follow-up reports, minimize information asymmetry between investors and companies, and improve the effectiveness of investor decision-making.

## Supporting information

S1 Appendix Details of data and methods. (DOCX)
