# Supplementary material for: Research on evolutionary game of environmental accounting information disclosure from the perspective of multi-agent
Source: PLoS One. 2021 Aug 31;16(8):e0256046. doi: 10.1371/journal.pone.0256046 (PMC8407557; doi:10.1371/journal.pone.0256046)
Supplement: S1 Appendix — (DOCX) [file pone.0256046.s001.docx]

**Research on evolutionary game of environmental accounting information disclosure from the perspective of multi-agent**

Yi’ang Qi, Jingjing Yao and Lindong Liu

**S1 Appendix. Details of data and methods**

# S1.1 Causality analysis

The main text has analyzed the causal feedback relationship in the model before constructing the system flow diagram. The causal feedback relationship diagram of the system dynamics model of the evolutionary game of enterprises, investors, and media is shown in S1 Figure.

**
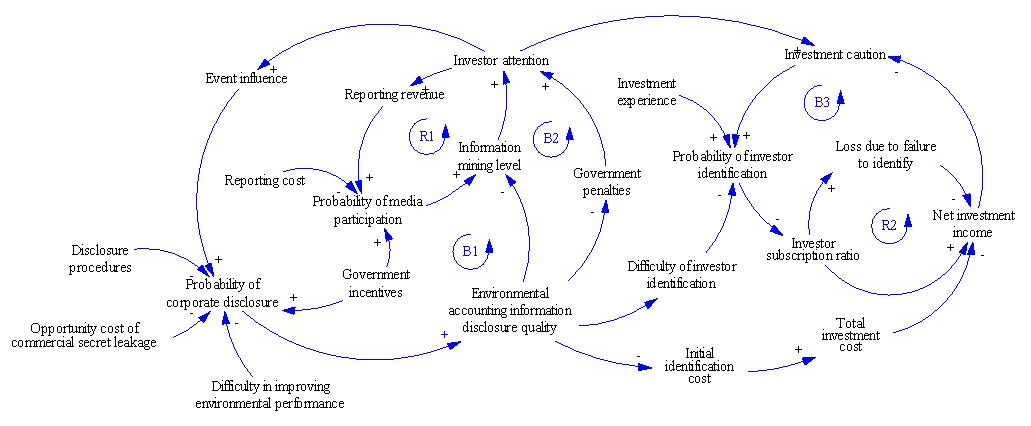
**

**S1 Figure. System dynamics model causal feedback relationship.**

1. **Loops of probability of corporate disclosure:**

The first one is B1: Probability of corporate disclosure⟶ Environmental accounting information disclosure quality⟶ Information mining level⟶ Investor attention⟶ Event influence (negative feedback);

With the decrease in the probability of corporate legal disclosure, the quality of environmental accounting information disclosure also declines. The decline in disclosure quality will increase media mining level of relevant information and media reporting behavior will increase investors’ attention to the company, and then the event influence will increase, which will lead to an increase in the probability of corporate disclosure.

The second one is B2: Probability of corporate disclosure⟶ Environmental accounting information disclosure quality⟶ Government penalties⟶ Investor attention⟶ Event influence (negative feedback);

With the decrease in the probability of corporate legal disclosure, the quality of environmental accounting information disclosure also declines. The decline in the quality of disclosure will cause the government to pay more attention to it, thereby increasing government penalties, and government actions will increase investors’ attention, and then the event influence will increase, which will lead to an increase in the probability of corporate disclosure.

1. **Loops of probability of media participation:**

R1: Probability of media participation⟶ Information mining level⟶ Investor attention⟶ Reporting revenue (positive feedback).

With the increase in the probability of media report, media information mining level on the quality of corporate environmental accounting information disclosure will also increase, which in turn makes investors pay more attention to corporate-related information, and the reporting revenue also increases, leading to the rise of the probability of media participation.

1. **Loops of probability of investor identification:**

The first one is R2: Probability of investor identification⟶ Investor subscription ratio⟶ Net investment income⟶ Investment caution (positive feedback);

As the probability of investor identification increases, the investor subscription ratio will decrease, and the net investment income will also decrease. Investment caution will continue to rise, leading to a further increase in the probability of investor identification.

The second one is B3: Probability of investor identification⟶ Investor subscription ratio⟶ Loss due to failure to identify⟶ Net investment income⟶ Investment caution (negative feedback);

As the probability of investor identification increases, the investor subscription ratio will decrease, and the investment loss caused by identification failure will also decrease, and the net income will increase. This makes the investment caution decline, leading to a reduction in the probability of investor identification.

# S1.2 Data sources

## S1.2.1 Initial simulation analysis

When the model is run at the initial value, the corresponding values of the probabilities of the three parties and the number of days are shown in S1 Table.

**S1 Table. The initial simulation results of the three parties.**

| **Time**  **(Day)** | **Probability of corporate disclosure** | **Probability of investor identification** | **Probability of media participation** |
| --- | --- | --- | --- |
| 1 | 0.30 | 0.30 | 0.40 |
| 2 | 0.29 | 0.38 | 0.35 |
| 3 | 0.27 | 0.42 | 0.33 |
| 4 | 0.26 | 0.45 | 0.31 |
| 5 | 0.25 | 0.46 | 0.30 |
| 6 | 0.24 | 0.47 | 0.28 |
| 7 | 0.23 | 0.48 | 0.27 |
| 8 | 0.23 | 0.49 | 0.27 |
| 9 | 0.22 | 0.49 | 0.26 |
| 10 | 0.21 | 0.50 | 0.25 |
| 11 | 0.20 | 0.50 | 0.24 |
| 12 | 0.20 | 0.50 | 0.24 |
| 13 | 0.19 | 0.51 | 0.23 |
| 14 | 0.18 | 0.51 | 0.23 |
| 15 | 0.18 | 0.51 | 0.22 |
| 16 | 0.17 | 0.51 | 0.22 |
| 17 | 0.17 | 0.51 | 0.21 |
| 18 | 0.16 | 0.51 | 0.21 |
| 19 | 0.16 | 0.51 | 0.21 |
| 20 | 0.16 | 0.51 | 0.20 |
| 21 | 0.15 | 0.51 | 0.20 |
| 22 | 0.15 | 0.51 | 0.20 |
| 23 | 0.14 | 0.51 | 0.19 |
| 24 | 0.14 | 0.52 | 0.19 |
| 25 | 0.14 | 0.52 | 0.19 |
| 26 | 0.13 | 0.52 | 0.19 |
| 27 | 0.13 | 0.52 | 0.18 |
| 28 | 0.13 | 0.52 | 0.18 |
| 29 | 0.12 | 0.52 | 0.18 |
| 30 | 0.12 | 0.52 | 0.18 |
| 31 | 0.12 | 0.52 | 0.17 |
| 32 | 0.12 | 0.52 | 0.17 |
| 33 | 0.11 | 0.52 | 0.17 |
| 34 | 0.11 | 0.52 | 0.17 |
| 35 | 0.11 | 0.52 | 0.16 |
| 36 | 0.11 | 0.52 | 0.16 |
| 37 | 0.10 | 0.52 | 0.16 |
| 38 | 0.10 | 0.53 | 0.16 |
| 39 | 0.10 | 0.53 | 0.16 |
| 40 | 0.10 | 0.53 | 0.16 |
| 41 | 0.10 | 0.53 | 0.15 |
| 42 | 0.09 | 0.53 | 0.15 |
| 43 | 0.09 | 0.53 | 0.15 |
| 44 | 0.09 | 0.53 | 0.15 |
| 45 | 0.09 | 0.53 | 0.15 |
| 46 | 0.09 | 0.53 | 0.15 |
| 47 | 0.09 | 0.53 | 0.14 |
| 48 | 0.08 | 0.53 | 0.14 |
| 49 | 0.08 | 0.53 | 0.14 |
| 50 | 0.08 | 0.54 | 0.14 |

## S1.2.2 Analysis of external variables in the main body of enterprise

1. Government incentives;

By changing the intensity of the government's incentives, it simulates the influence of the government's different levels of incentives on the strategic choices of the tripartite entities. The government incentive is increased by 10% on the basis of the initial value, that is, set to 0.4, and the simulation results of the corresponding three-party evolutionary game are shown in S2 Table.

**S2 Table. When the government incentive is 0.4.**

| **Time**  **(Day)** | **Probability of corporate disclosure** | **Probability of investor identification** | **Probability of media participation** |
| --- | --- | --- | --- |
| 1 | 0.30 | 0.30 | 0.40 |
| 2 | 0.32 | 0.38 | 0.39 |
| 3 | 0.33 | 0.42 | 0.39 |
| 4 | 0.34 | 0.43 | 0.39 |
| 5 | 0.35 | 0.44 | 0.39 |
| 6 | 0.36 | 0.43 | 0.39 |
| 7 | 0.37 | 0.42 | 0.39 |
| 8 | 0.38 | 0.41 | 0.39 |
| 9 | 0.39 | 0.40 | 0.39 |
| 10 | 0.40 | 0.39 | 0.39 |
| 11 | 0.40 | 0.38 | 0.39 |
| 12 | 0.41 | 0.36 | 0.40 |
| 13 | 0.42 | 0.35 | 0.40 |
| 14 | 0.42 | 0.34 | 0.40 |
| 15 | 0.43 | 0.33 | 0.40 |
| 16 | 0.44 | 0.32 | 0.40 |
| 17 | 0.44 | 0.30 | 0.40 |
| 18 | 0.45 | 0.29 | 0.40 |
| 19 | 0.45 | 0.28 | 0.40 |
| 20 | 0.46 | 0.27 | 0.40 |
| 21 | 0.46 | 0.26 | 0.40 |
| 22 | 0.47 | 0.25 | 0.40 |
| 23 | 0.47 | 0.24 | 0.40 |
| 24 | 0.47 | 0.23 | 0.40 |
| 25 | 0.48 | 0.22 | 0.41 |
| 26 | 0.48 | 0.22 | 0.41 |
| 27 | 0.48 | 0.21 | 0.41 |
| 28 | 0.49 | 0.20 | 0.41 |
| 29 | 0.49 | 0.19 | 0.41 |
| 30 | 0.49 | 0.19 | 0.41 |
| 31 | 0.50 | 0.18 | 0.41 |
| 32 | 0.50 | 0.17 | 0.41 |
| 33 | 0.50 | 0.18 | 0.41 |
| 34 | 0.50 | 0.18 | 0.41 |
| 35 | 0.50 | 0.18 | 0.41 |
| 36 | 0.50 | 0.18 | 0.41 |
| 37 | 0.50 | 0.19 | 0.41 |
| 38 | 0.50 | 0.19 | 0.41 |
| 39 | 0.51 | 0.19 | 0.41 |
| 40 | 0.51 | 0.19 | 0.41 |
| 41 | 0.51 | 0.20 | 0.41 |
| 42 | 0.51 | 0.20 | 0.41 |
| 43 | 0.51 | 0.20 | 0.41 |
| 44 | 0.51 | 0.20 | 0.41 |
| 45 | 0.51 | 0.20 | 0.41 |
| 46 | 0.51 | 0.21 | 0.41 |
| 47 | 0.51 | 0.21 | 0.41 |
| 48 | 0.51 | 0.21 | 0.41 |
| 49 | 0.51 | 0.21 | 0.41 |
| 50 | 0.51 | 0.21 | 0.41 |

The government incentives are increased by 20% on the basis of the initial value, that is, set to 0.5, and the simulation results of the corresponding tripartite evolutionary game are shown in S3 Table.

**S3 Table. When the government incentive is 0.5.**

| **Time**  **(Day)** | **Probability of corporate disclosure** | **Probability of investor identification** | **Probability of media participation** |
| --- | --- | --- | --- |
| 1 | 0.30 | 0.30 | 0.40 |
| 2 | 0.35 | 0.38 | 0.43 |
| 3 | 0.39 | 0.38 | 0.45 |
| 4 | 0.42 | 0.35 | 0.47 |
| 5 | 0.45 | 0.30 | 0.48 |
| 6 | 0.48 | 0.26 | 0.50 |
| 7 | 0.50 | 0.23 | 0.51 |
| 8 | 0.51 | 0.24 | 0.51 |
| 9 | 0.52 | 0.25 | 0.52 |
| 10 | 0.53 | 0.26 | 0.52 |
| 11 | 0.53 | 0.27 | 0.52 |
| 12 | 0.54 | 0.28 | 0.53 |
| 13 | 0.54 | 0.29 | 0.53 |
| 14 | 0.55 | 0.29 | 0.53 |
| 15 | 0.55 | 0.30 | 0.54 |
| 16 | 0.56 | 0.31 | 0.54 |
| 17 | 0.56 | 0.31 | 0.54 |
| 18 | 0.56 | 0.32 | 0.54 |
| 19 | 0.57 | 0.32 | 0.54 |
| 20 | 0.57 | 0.33 | 0.55 |
| 21 | 0.57 | 0.33 | 0.55 |
| 22 | 0.57 | 0.33 | 0.55 |
| 23 | 0.58 | 0.34 | 0.55 |
| 24 | 0.58 | 0.34 | 0.55 |
| 25 | 0.58 | 0.34 | 0.55 |
| 26 | 0.58 | 0.35 | 0.56 |
| 27 | 0.59 | 0.35 | 0.56 |
| 28 | 0.59 | 0.35 | 0.56 |
| 29 | 0.59 | 0.36 | 0.56 |
| 30 | 0.59 | 0.36 | 0.56 |
| 31 | 0.59 | 0.36 | 0.56 |
| 32 | 0.60 | 0.36 | 0.56 |
| 33 | 0.60 | 0.37 | 0.56 |
| 34 | 0.60 | 0.37 | 0.57 |
| 35 | 0.60 | 0.37 | 0.57 |
| 36 | 0.60 | 0.37 | 0.57 |
| 37 | 0.60 | 0.38 | 0.57 |
| 38 | 0.61 | 0.38 | 0.57 |
| 39 | 0.61 | 0.38 | 0.57 |
| 40 | 0.61 | 0.38 | 0.57 |
| 41 | 0.61 | 0.39 | 0.57 |
| 42 | 0.61 | 0.39 | 0.57 |
| 43 | 0.61 | 0.39 | 0.57 |
| 44 | 0.61 | 0.39 | 0.57 |
| 45 | 0.61 | 0.39 | 0.57 |
| 46 | 0.62 | 0.39 | 0.58 |
| 47 | 0.62 | 0.40 | 0.58 |
| 48 | 0.62 | 0.40 | 0.58 |
| 49 | 0.62 | 0.40 | 0.58 |
| 50 | 0.62 | 0.40 | 0.58 |

2. The degree of cumbersome disclosure procedures;

By changing the value of the cumbersome degree of the disclosure procedure, the impact of the disclosure procedure on the strategic choices of the three parties is simulated. The cumbersomeness of the disclosure procedure is reduced by 10% on the basis of the initial value, that is, set to 0.5, and the three-party evolutionary game results corresponding to the simulation are shown in S4 Table.

**S4 Table. When the cumbersomeness of the disclosure procedure is 0.5.**

| **Time**  **(Day)** | **Probability of corporate disclosure** | **Probability of investor identification** | **Probability of media participation** |
| --- | --- | --- | --- |
| 1 | 0.30 | 0.30 | 0.40 |
| 2 | 0.31 | 0.38 | 0.35 |
| 3 | 0.31 | 0.43 | 0.33 |
| 4 | 0.31 | 0.45 | 0.31 |
| 5 | 0.31 | 0.47 | 0.30 |
| 6 | 0.32 | 0.49 | 0.29 |
| 7 | 0.32 | 0.50 | 0.28 |
| 8 | 0.32 | 0.51 | 0.28 |
| 9 | 0.32 | 0.52 | 0.27 |
| 10 | 0.32 | 0.53 | 0.27 |
| 11 | 0.32 | 0.54 | 0.26 |
| 12 | 0.32 | 0.55 | 0.26 |
| 13 | 0.32 | 0.55 | 0.25 |
| 14 | 0.32 | 0.56 | 0.25 |
| 15 | 0.32 | 0.56 | 0.25 |
| 16 | 0.32 | 0.56 | 0.24 |
| 17 | 0.32 | 0.57 | 0.24 |
| 18 | 0.32 | 0.57 | 0.24 |
| 19 | 0.32 | 0.58 | 0.23 |
| 20 | 0.32 | 0.58 | 0.23 |
| 21 | 0.32 | 0.58 | 0.23 |
| 22 | 0.32 | 0.58 | 0.23 |
| 23 | 0.32 | 0.59 | 0.22 |
| 24 | 0.32 | 0.59 | 0.22 |
| 25 | 0.32 | 0.59 | 0.22 |
| 26 | 0.32 | 0.59 | 0.22 |
| 27 | 0.32 | 0.60 | 0.22 |
| 28 | 0.32 | 0.60 | 0.21 |
| 29 | 0.32 | 0.60 | 0.21 |
| 30 | 0.32 | 0.60 | 0.21 |
| 31 | 0.32 | 0.60 | 0.21 |
| 32 | 0.32 | 0.61 | 0.21 |
| 33 | 0.32 | 0.61 | 0.21 |
| 34 | 0.32 | 0.61 | 0.20 |
| 35 | 0.32 | 0.61 | 0.20 |
| 36 | 0.32 | 0.61 | 0.20 |
| 37 | 0.32 | 0.61 | 0.20 |
| 38 | 0.32 | 0.62 | 0.20 |
| 39 | 0.32 | 0.62 | 0.20 |
| 40 | 0.32 | 0.62 | 0.20 |
| 41 | 0.32 | 0.62 | 0.20 |
| 42 | 0.32 | 0.62 | 0.19 |
| 43 | 0.32 | 0.62 | 0.19 |
| 44 | 0.32 | 0.62 | 0.19 |
| 45 | 0.32 | 0.63 | 0.19 |
| 46 | 0.32 | 0.63 | 0.19 |
| 47 | 0.32 | 0.63 | 0.19 |
| 48 | 0.32 | 0.63 | 0.19 |
| 49 | 0.32 | 0.63 | 0.19 |
| 50 | 0.32 | 0.63 | 0.19 |

The cumbersomeness of the disclosure procedure is reduced by 20% on the basis of the initial value, that is, set to 0.4, and the three-party evolutionary game results corresponding to the simulation are shown in S5 Table.

**S5 Table. When the cumbersomeness of the disclosure procedure is 0.4.**

| **Time**  **(Day)** | **Probability of corporate disclosure** | **Probability of investor identification** | **Probability of media participation** |
| --- | --- | --- | --- |
| 1 | 0.30 | 0.30 | 0.40 |
| 2 | 0.33 | 0.38 | 0.35 |
| 3 | 0.35 | 0.41 | 0.33 |
| 4 | 0.36 | 0.41 | 0.32 |
| 5 | 0.38 | 0.39 | 0.31 |
| 6 | 0.39 | 0.38 | 0.30 |
| 7 | 0.40 | 0.36 | 0.29 |
| 8 | 0.41 | 0.34 | 0.29 |
| 9 | 0.42 | 0.32 | 0.29 |
| 10 | 0.43 | 0.30 | 0.28 |
| 11 | 0.44 | 0.28 | 0.28 |
| 12 | 0.45 | 0.26 | 0.28 |
| 13 | 0.46 | 0.24 | 0.27 |
| 14 | 0.46 | 0.23 | 0.27 |
| 15 | 0.47 | 0.21 | 0.27 |
| 16 | 0.48 | 0.20 | 0.27 |
| 17 | 0.48 | 0.19 | 0.27 |
| 18 | 0.49 | 0.18 | 0.26 |
| 19 | 0.49 | 0.16 | 0.26 |
| 20 | 0.50 | 0.15 | 0.26 |
| 21 | 0.50 | 0.14 | 0.26 |
| 22 | 0.50 | 0.13 | 0.26 |
| 23 | 0.50 | 0.14 | 0.26 |
| 24 | 0.51 | 0.14 | 0.25 |
| 25 | 0.51 | 0.15 | 0.25 |
| 26 | 0.51 | 0.15 | 0.25 |
| 27 | 0.51 | 0.15 | 0.25 |
| 28 | 0.51 | 0.16 | 0.25 |
| 29 | 0.51 | 0.16 | 0.24 |
| 30 | 0.51 | 0.16 | 0.24 |
| 31 | 0.51 | 0.16 | 0.24 |
| 32 | 0.51 | 0.17 | 0.24 |
| 33 | 0.51 | 0.17 | 0.24 |
| 34 | 0.51 | 0.17 | 0.24 |
| 35 | 0.51 | 0.17 | 0.23 |
| 36 | 0.51 | 0.18 | 0.23 |
| 37 | 0.51 | 0.18 | 0.23 |
| 38 | 0.51 | 0.18 | 0.23 |
| 39 | 0.51 | 0.18 | 0.23 |
| 40 | 0.51 | 0.19 | 0.23 |
| 41 | 0.52 | 0.19 | 0.23 |
| 42 | 0.52 | 0.19 | 0.23 |
| 43 | 0.52 | 0.19 | 0.22 |
| 44 | 0.52 | 0.19 | 0.22 |
| 45 | 0.52 | 0.19 | 0.22 |
| 46 | 0.52 | 0.20 | 0.22 |
| 47 | 0.52 | 0.20 | 0.22 |
| 48 | 0.52 | 0.20 | 0.22 |
| 49 | 0.52 | 0.20 | 0.22 |
| 50 | 0.52 | 0.20 | 0.22 |

3. Difficulty in improving environmental performance;

By changing the value of the difficulty of environmental performance improvement, the impact of the difficulty of improving environmental performance on the strategic choices of the three parties is simulated. The difficulty of improving environmental performance is reduced by 10%, on the basis of the initial value, that is, set to 0.8. The three-party evolutionary game results corresponding to the simulation are shown in S6 Table.

**S6 Table. When the environmental performance improvement difficulty is 0.8.**

| **Time**  **(Day)** | **Probability of corporate disclosure** | **Probability of investor identification** | **Probability of media participation** |
| --- | --- | --- | --- |
| 1 | 0.30 | 0.30 | 0.40 |
| 2 | 0.31 | 0.38 | 0.35 |
| 3 | 0.31 | 0.43 | 0.33 |
| 4 | 0.31 | 0.45 | 0.31 |
| 5 | 0.31 | 0.47 | 0.30 |
| 6 | 0.32 | 0.49 | 0.29 |
| 7 | 0.32 | 0.50 | 0.28 |
| 8 | 0.32 | 0.51 | 0.28 |
| 9 | 0.32 | 0.52 | 0.27 |
| 10 | 0.32 | 0.53 | 0.27 |
| 11 | 0.32 | 0.54 | 0.26 |
| 12 | 0.32 | 0.55 | 0.26 |
| 13 | 0.32 | 0.55 | 0.25 |
| 14 | 0.32 | 0.56 | 0.25 |
| 15 | 0.32 | 0.56 | 0.25 |
| 16 | 0.32 | 0.56 | 0.24 |
| 17 | 0.32 | 0.57 | 0.24 |
| 18 | 0.32 | 0.57 | 0.24 |
| 19 | 0.32 | 0.58 | 0.23 |
| 20 | 0.32 | 0.58 | 0.23 |
| 21 | 0.32 | 0.58 | 0.23 |
| 22 | 0.32 | 0.58 | 0.23 |
| 23 | 0.32 | 0.59 | 0.22 |
| 24 | 0.32 | 0.59 | 0.22 |
| 25 | 0.32 | 0.59 | 0.22 |
| 26 | 0.32 | 0.59 | 0.22 |
| 27 | 0.32 | 0.60 | 0.22 |
| 28 | 0.32 | 0.60 | 0.21 |
| 29 | 0.32 | 0.60 | 0.21 |
| 30 | 0.32 | 0.60 | 0.21 |
| 31 | 0.32 | 0.60 | 0.21 |
| 32 | 0.32 | 0.61 | 0.21 |
| 33 | 0.32 | 0.61 | 0.21 |
| 34 | 0.32 | 0.61 | 0.20 |
| 35 | 0.32 | 0.61 | 0.20 |
| 36 | 0.32 | 0.61 | 0.20 |
| 37 | 0.32 | 0.61 | 0.20 |
| 38 | 0.32 | 0.62 | 0.20 |
| 39 | 0.32 | 0.62 | 0.20 |
| 40 | 0.32 | 0.62 | 0.20 |
| 41 | 0.32 | 0.62 | 0.20 |
| 42 | 0.32 | 0.62 | 0.19 |
| 43 | 0.32 | 0.62 | 0.19 |
| 44 | 0.32 | 0.62 | 0.19 |
| 45 | 0.32 | 0.63 | 0.19 |
| 46 | 0.32 | 0.63 | 0.19 |
| 47 | 0.32 | 0.63 | 0.19 |
| 48 | 0.32 | 0.63 | 0.19 |
| 49 | 0.32 | 0.63 | 0.19 |
| 50 | 0.32 | 0.63 | 0.19 |

The difficulty of improving environmental performance is reduced by 20%, on the basis of the initial value, that is, set to 0.7. The three-party evolutionary game results corresponding to the simulation are shown in S7 Table.

**S7 Table. When the environmental performance improvement difficulty is 0.7.**

| **Time**  **(Day)** | **Probability of corporate disclosure** | **Probability of investor identification** | **Probability of media participation** |
| --- | --- | --- | --- |
| 1 | 0.30 | 0.30 | 0.40 |
| 2 | 0.33 | 0.38 | 0.35 |
| 3 | 0.35 | 0.41 | 0.33 |
| 4 | 0.36 | 0.41 | 0.32 |
| 5 | 0.38 | 0.39 | 0.31 |
| 6 | 0.39 | 0.38 | 0.30 |
| 7 | 0.40 | 0.36 | 0.29 |
| 8 | 0.41 | 0.34 | 0.29 |
| 9 | 0.42 | 0.32 | 0.29 |
| 10 | 0.43 | 0.30 | 0.28 |
| 11 | 0.44 | 0.28 | 0.28 |
| 12 | 0.45 | 0.26 | 0.28 |
| 13 | 0.46 | 0.24 | 0.27 |
| 14 | 0.46 | 0.23 | 0.27 |
| 15 | 0.47 | 0.21 | 0.27 |
| 16 | 0.48 | 0.20 | 0.27 |
| 17 | 0.48 | 0.19 | 0.27 |
| 18 | 0.49 | 0.18 | 0.26 |
| 19 | 0.49 | 0.16 | 0.26 |
| 20 | 0.50 | 0.15 | 0.26 |
| 21 | 0.50 | 0.14 | 0.26 |
| 22 | 0.50 | 0.13 | 0.26 |
| 23 | 0.50 | 0.14 | 0.26 |
| 24 | 0.51 | 0.14 | 0.25 |
| 25 | 0.51 | 0.15 | 0.25 |
| 26 | 0.51 | 0.15 | 0.25 |
| 27 | 0.51 | 0.15 | 0.25 |
| 28 | 0.51 | 0.16 | 0.25 |
| 29 | 0.51 | 0.16 | 0.24 |
| 30 | 0.51 | 0.16 | 0.24 |
| 31 | 0.51 | 0.16 | 0.24 |
| 32 | 0.51 | 0.17 | 0.24 |
| 33 | 0.51 | 0.17 | 0.24 |
| 34 | 0.51 | 0.17 | 0.24 |
| 35 | 0.51 | 0.17 | 0.23 |
| 36 | 0.51 | 0.18 | 0.23 |
| 37 | 0.51 | 0.18 | 0.23 |
| 38 | 0.51 | 0.18 | 0.23 |
| 39 | 0.51 | 0.18 | 0.23 |
| 40 | 0.51 | 0.19 | 0.23 |
| 41 | 0.52 | 0.19 | 0.23 |
| 42 | 0.52 | 0.19 | 0.23 |
| 43 | 0.52 | 0.19 | 0.22 |
| 44 | 0.52 | 0.19 | 0.22 |
| 45 | 0.52 | 0.19 | 0.22 |
| 46 | 0.52 | 0.20 | 0.22 |
| 47 | 0.52 | 0.20 | 0.22 |
| 48 | 0.52 | 0.20 | 0.22 |
| 49 | 0.52 | 0.20 | 0.22 |
| 50 | 0.52 | 0.20 | 0.22 |

4. Proportion of opportunity cost of commercial secret leakage;

By changing the proportion of the opportunity cost of the leakage of trade secrets, the impact of the opportunity cost of the leakage of trade secrets on the strategic choices of the three parties is simulated. Decreasing the opportunity cost ratio by 10% on the basis of the initial value, that is, setting it to 0.4, the three-party evolutionary game results corresponding to the simulation are shown in S8 Table.

**S8 Table. When the opportunity cost ratio is 0.4.**

| **Time**  **(Day)** | **Probability of corporate disclosure** | **Probability of investor identification** | **Probability of media participation** |
| --- | --- | --- | --- |
| 1 | 0.30 | 0.30 | 0.40 |
| 2 | 0.30 | 0.38 | 0.35 |
| 3 | 0.29 | 0.42 | 0.33 |
| 4 | 0.29 | 0.45 | 0.31 |
| 5 | 0.28 | 0.47 | 0.30 |
| 6 | 0.28 | 0.48 | 0.29 |
| 7 | 0.27 | 0.50 | 0.28 |
| 8 | 0.27 | 0.51 | 0.27 |
| 9 | 0.27 | 0.51 | 0.26 |
| 10 | 0.26 | 0.52 | 0.26 |
| 11 | 0.26 | 0.53 | 0.25 |
| 12 | 0.25 | 0.53 | 0.25 |
| 13 | 0.25 | 0.54 | 0.24 |
| 14 | 0.25 | 0.54 | 0.24 |
| 15 | 0.24 | 0.55 | 0.23 |
| 16 | 0.24 | 0.55 | 0.23 |
| 17 | 0.24 | 0.55 | 0.22 |
| 18 | 0.23 | 0.56 | 0.22 |
| 19 | 0.23 | 0.56 | 0.22 |
| 20 | 0.23 | 0.56 | 0.21 |
| 21 | 0.23 | 0.56 | 0.21 |
| 22 | 0.22 | 0.56 | 0.21 |
| 23 | 0.22 | 0.57 | 0.21 |
| 24 | 0.22 | 0.57 | 0.20 |
| 25 | 0.22 | 0.57 | 0.20 |
| 26 | 0.21 | 0.57 | 0.20 |
| 27 | 0.21 | 0.57 | 0.19 |
| 28 | 0.21 | 0.57 | 0.19 |
| 29 | 0.21 | 0.57 | 0.19 |
| 30 | 0.20 | 0.57 | 0.19 |
| 31 | 0.20 | 0.58 | 0.19 |
| 32 | 0.20 | 0.58 | 0.18 |
| 33 | 0.20 | 0.58 | 0.18 |
| 34 | 0.19 | 0.58 | 0.18 |
| 35 | 0.19 | 0.58 | 0.18 |
| 36 | 0.19 | 0.58 | 0.17 |
| 37 | 0.19 | 0.58 | 0.17 |
| 38 | 0.19 | 0.58 | 0.17 |
| 39 | 0.18 | 0.58 | 0.17 |
| 40 | 0.18 | 0.58 | 0.17 |
| 41 | 0.18 | 0.58 | 0.17 |
| 42 | 0.18 | 0.58 | 0.16 |
| 43 | 0.18 | 0.58 | 0.16 |
| 44 | 0.18 | 0.58 | 0.16 |
| 45 | 0.17 | 0.58 | 0.16 |
| 46 | 0.17 | 0.58 | 0.16 |
| 47 | 0.17 | 0.58 | 0.16 |
| 48 | 0.17 | 0.58 | 0.16 |
| 49 | 0.17 | 0.58 | 0.15 |
| 50 | 0.17 | 0.58 | 0.15 |

Decreasing the opportunity cost ratio by 20% on the basis of the initial value, that is, setting it to 0.3, the three-party evolutionary game results corresponding to the simulation are shown in S9 Table.

**S9 Table. When the opportunity cost ratio is 0.3.**

| **Time**  **(Day)** | **Probability of corporate disclosure** | **Probability of investor identification** | **Probability of media participation** |
| --- | --- | --- | --- |
| 1 | 0.30 | 0.30 | 0.40 |
| 2 | 0.31 | 0.38 | 0.35 |
| 3 | 0.31 | 0.43 | 0.33 |
| 4 | 0.31 | 0.45 | 0.31 |
| 5 | 0.31 | 0.47 | 0.30 |
| 6 | 0.32 | 0.49 | 0.29 |
| 7 | 0.32 | 0.50 | 0.28 |
| 8 | 0.32 | 0.51 | 0.28 |
| 9 | 0.32 | 0.52 | 0.27 |
| 10 | 0.32 | 0.53 | 0.27 |
| 11 | 0.32 | 0.54 | 0.26 |
| 12 | 0.32 | 0.55 | 0.26 |
| 13 | 0.32 | 0.55 | 0.25 |
| 14 | 0.32 | 0.56 | 0.25 |
| 15 | 0.32 | 0.56 | 0.25 |
| 16 | 0.32 | 0.56 | 0.24 |
| 17 | 0.32 | 0.57 | 0.24 |
| 18 | 0.32 | 0.57 | 0.24 |
| 19 | 0.32 | 0.58 | 0.23 |
| 20 | 0.32 | 0.58 | 0.23 |
| 21 | 0.32 | 0.58 | 0.23 |
| 22 | 0.32 | 0.58 | 0.23 |
| 23 | 0.32 | 0.59 | 0.22 |
| 24 | 0.32 | 0.59 | 0.22 |
| 25 | 0.32 | 0.59 | 0.22 |
| 26 | 0.32 | 0.59 | 0.22 |
| 27 | 0.32 | 0.60 | 0.22 |
| 28 | 0.32 | 0.60 | 0.21 |
| 29 | 0.32 | 0.60 | 0.21 |
| 30 | 0.32 | 0.60 | 0.21 |
| 31 | 0.32 | 0.60 | 0.21 |
| 32 | 0.32 | 0.61 | 0.21 |
| 33 | 0.32 | 0.61 | 0.21 |
| 34 | 0.32 | 0.61 | 0.20 |
| 35 | 0.32 | 0.61 | 0.20 |
| 36 | 0.32 | 0.61 | 0.20 |
| 37 | 0.32 | 0.61 | 0.20 |
| 38 | 0.32 | 0.62 | 0.20 |
| 39 | 0.32 | 0.62 | 0.20 |
| 40 | 0.32 | 0.62 | 0.20 |
| 41 | 0.32 | 0.62 | 0.20 |
| 42 | 0.32 | 0.62 | 0.19 |
| 43 | 0.32 | 0.62 | 0.19 |
| 44 | 0.32 | 0.62 | 0.19 |
| 45 | 0.32 | 0.63 | 0.19 |
| 46 | 0.32 | 0.63 | 0.19 |
| 47 | 0.32 | 0.63 | 0.19 |
| 48 | 0.32 | 0.63 | 0.19 |
| 49 | 0.32 | 0.63 | 0.19 |
| 50 | 0.32 | 0.63 | 0.19 |

## S1.2.3 Analysis of external variables in the main body of investors

By changing the level of investment experience, it simulates the impact of the level of investment experience on the strategic choices of the tripartite entities. Increase the experience level by 10% on the basis of the initial value, that is, set to 0.2, then the three-way evolutionary game results corresponding to the simulation are shown in S10 Table.

**S10 Table. When the investment experience level is 0.2.**

| **Time**  **(Day)** | **Probability of corporate disclosure** | **Probability of investor identification** | **Probability of media participation** |
| --- | --- | --- | --- |
| 1 | 0.30 | 0.30 | 0.40 |
| 2 | 0.29 | 0.42 | 0.35 |
| 3 | 0.27 | 0.48 | 0.33 |
| 4 | 0.26 | 0.52 | 0.31 |
| 5 | 0.25 | 0.55 | 0.30 |
| 6 | 0.24 | 0.56 | 0.28 |
| 7 | 0.23 | 0.58 | 0.27 |
| 8 | 0.23 | 0.59 | 0.27 |
| 9 | 0.22 | 0.60 | 0.26 |
| 10 | 0.21 | 0.61 | 0.25 |
| 11 | 0.20 | 0.62 | 0.24 |
| 12 | 0.20 | 0.62 | 0.24 |
| 13 | 0.19 | 0.63 | 0.23 |
| 14 | 0.18 | 0.63 | 0.23 |
| 15 | 0.18 | 0.64 | 0.22 |
| 16 | 0.17 | 0.64 | 0.22 |
| 17 | 0.17 | 0.64 | 0.21 |
| 18 | 0.16 | 0.65 | 0.21 |
| 19 | 0.16 | 0.65 | 0.21 |
| 20 | 0.16 | 0.65 | 0.20 |
| 21 | 0.15 | 0.66 | 0.20 |
| 22 | 0.15 | 0.66 | 0.20 |
| 23 | 0.14 | 0.66 | 0.19 |
| 24 | 0.14 | 0.66 | 0.19 |
| 25 | 0.14 | 0.67 | 0.19 |
| 26 | 0.13 | 0.67 | 0.19 |
| 27 | 0.13 | 0.67 | 0.18 |
| 28 | 0.13 | 0.67 | 0.18 |
| 29 | 0.12 | 0.67 | 0.18 |
| 30 | 0.12 | 0.68 | 0.18 |
| 31 | 0.12 | 0.68 | 0.17 |
| 32 | 0.12 | 0.68 | 0.17 |
| 33 | 0.11 | 0.68 | 0.17 |
| 34 | 0.11 | 0.68 | 0.17 |
| 35 | 0.11 | 0.69 | 0.16 |
| 36 | 0.11 | 0.69 | 0.16 |
| 37 | 0.10 | 0.69 | 0.16 |
| 38 | 0.10 | 0.69 | 0.16 |
| 39 | 0.10 | 0.69 | 0.16 |
| 40 | 0.10 | 0.70 | 0.16 |
| 41 | 0.10 | 0.70 | 0.15 |
| 42 | 0.09 | 0.70 | 0.15 |
| 43 | 0.09 | 0.70 | 0.15 |
| 44 | 0.09 | 0.70 | 0.15 |
| 45 | 0.09 | 0.70 | 0.15 |
| 46 | 0.09 | 0.71 | 0.15 |
| 47 | 0.09 | 0.71 | 0.14 |
| 48 | 0.08 | 0.71 | 0.14 |
| 49 | 0.08 | 0.71 | 0.14 |
| 50 | 0.08 | 0.71 | 0.14 |

Increase the experience level by 20% on the basis of the initial value, that is, set to 0.3, then the three-way evolutionary game results corresponding to the simulation are shown in S11 Table.

**S11 Table. When the investment experience level is 0.3.**

| **Time**  **(Day)** | **Probability of corporate disclosure** | **Probability of investor identification** | **Probability of media participation** |
| --- | --- | --- | --- |
| 1 | 0.30 | 0.30 | 0.40 |
| 2 | 0.29 | 0.46 | 0.35 |
| 3 | 0.27 | 0.54 | 0.33 |
| 4 | 0.26 | 0.59 | 0.31 |
| 5 | 0.25 | 0.63 | 0.30 |
| 6 | 0.24 | 0.66 | 0.28 |
| 7 | 0.23 | 0.68 | 0.27 |
| 8 | 0.23 | 0.70 | 0.27 |
| 9 | 0.22 | 0.71 | 0.26 |
| 10 | 0.21 | 0.72 | 0.25 |
| 11 | 0.20 | 0.73 | 0.24 |
| 12 | 0.20 | 0.74 | 0.24 |
| 13 | 0.19 | 0.75 | 0.23 |
| 14 | 0.18 | 0.76 | 0.23 |
| 15 | 0.18 | 0.77 | 0.22 |
| 16 | 0.17 | 0.77 | 0.22 |
| 17 | 0.17 | 0.78 | 0.21 |
| 18 | 0.16 | 0.79 | 0.21 |
| 19 | 0.16 | 0.79 | 0.21 |
| 20 | 0.16 | 0.80 | 0.20 |
| 21 | 0.15 | 0.80 | 0.20 |
| 22 | 0.15 | 0.80 | 0.20 |
| 23 | 0.14 | 0.81 | 0.19 |
| 24 | 0.14 | 0.81 | 0.19 |
| 25 | 0.14 | 0.82 | 0.19 |
| 26 | 0.13 | 0.82 | 0.19 |
| 27 | 0.13 | 0.82 | 0.18 |
| 28 | 0.13 | 0.83 | 0.18 |
| 29 | 0.12 | 0.83 | 0.18 |
| 30 | 0.12 | 0.83 | 0.18 |
| 31 | 0.12 | 0.84 | 0.17 |
| 32 | 0.12 | 0.84 | 0.17 |
| 33 | 0.11 | 0.84 | 0.17 |
| 34 | 0.11 | 0.85 | 0.17 |
| 35 | 0.11 | 0.85 | 0.16 |
| 36 | 0.11 | 0.85 | 0.16 |
| 37 | 0.10 | 0.86 | 0.16 |
| 38 | 0.10 | 0.86 | 0.16 |
| 39 | 0.10 | 0.86 | 0.16 |
| 40 | 0.10 | 0.86 | 0.16 |
| 41 | 0.10 | 0.87 | 0.15 |
| 42 | 0.09 | 0.87 | 0.15 |
| 43 | 0.09 | 0.87 | 0.15 |
| 44 | 0.09 | 0.88 | 0.15 |
| 45 | 0.09 | 0.88 | 0.15 |
| 46 | 0.09 | 0.88 | 0.15 |
| 47 | 0.09 | 0.88 | 0.14 |
| 48 | 0.08 | 0.89 | 0.14 |
| 49 | 0.08 | 0.89 | 0.14 |
| 50 | 0.08 | 0.89 | 0.14 |

## S1.2.4 Analysis of external variables in the main body of the media

By changing the ratio of media reporting costs, it simulates the impact of media reporting costs on the strategic choices of tripartite entities. The media report cost ratio is reduced by 10% on the basis of the initial value, that is, set to 0.5. The three-party evolutionary game results corresponding to the simulation are shown in S12 Table.

**S12 Table. When the media report cost ratio is 0.5.**

| **Time**  **(Day)** | **Probability of corporate disclosure** | **Probability of investor identification** | **Probability of media participation** |
| --- | --- | --- | --- |
| 1 | 0.30 | 0.30 | 0.40 |
| 2 | 0.29 | 0.38 | 0.39 |
| 3 | 0.28 | 0.42 | 0.39 |
| 4 | 0.27 | 0.45 | 0.38 |
| 5 | 0.26 | 0.46 | 0.38 |
| 6 | 0.25 | 0.48 | 0.38 |
| 7 | 0.25 | 0.49 | 0.38 |
| 8 | 0.24 | 0.49 | 0.37 |
| 9 | 0.23 | 0.50 | 0.37 |
| 10 | 0.23 | 0.51 | 0.37 |
| 11 | 0.22 | 0.51 | 0.37 |
| 12 | 0.22 | 0.51 | 0.37 |
| 13 | 0.21 | 0.52 | 0.36 |
| 14 | 0.21 | 0.52 | 0.36 |
| 15 | 0.20 | 0.52 | 0.36 |
| 16 | 0.20 | 0.52 | 0.36 |
| 17 | 0.20 | 0.53 | 0.36 |
| 18 | 0.19 | 0.53 | 0.36 |
| 19 | 0.19 | 0.53 | 0.36 |
| 20 | 0.19 | 0.53 | 0.35 |
| 21 | 0.18 | 0.53 | 0.35 |
| 22 | 0.18 | 0.53 | 0.35 |
| 23 | 0.18 | 0.53 | 0.35 |
| 24 | 0.17 | 0.53 | 0.35 |
| 25 | 0.17 | 0.53 | 0.35 |
| 26 | 0.17 | 0.54 | 0.35 |
| 27 | 0.17 | 0.54 | 0.35 |
| 28 | 0.16 | 0.54 | 0.35 |
| 29 | 0.16 | 0.54 | 0.35 |
| 30 | 0.16 | 0.54 | 0.34 |
| 31 | 0.16 | 0.54 | 0.34 |
| 32 | 0.16 | 0.54 | 0.34 |
| 33 | 0.15 | 0.54 | 0.34 |
| 34 | 0.15 | 0.54 | 0.34 |
| 35 | 0.15 | 0.54 | 0.34 |
| 36 | 0.15 | 0.54 | 0.34 |
| 37 | 0.15 | 0.54 | 0.34 |
| 38 | 0.14 | 0.54 | 0.34 |
| 39 | 0.14 | 0.54 | 0.34 |
| 40 | 0.14 | 0.54 | 0.34 |
| 41 | 0.14 | 0.54 | 0.34 |
| 42 | 0.14 | 0.54 | 0.34 |
| 43 | 0.14 | 0.54 | 0.34 |
| 44 | 0.13 | 0.55 | 0.34 |
| 45 | 0.13 | 0.55 | 0.34 |
| 46 | 0.13 | 0.55 | 0.33 |
| 47 | 0.13 | 0.55 | 0.33 |
| 48 | 0.13 | 0.55 | 0.33 |
| 49 | 0.13 | 0.55 | 0.33 |
| 50 | 0.12 | 0.55 | 0.33 |

The media report cost ratio is reduced by 20% on the basis of the initial value, that is, set to 0.4. The three-party evolutionary game results corresponding to the simulation are shown in S13 Table.

**S13 Table. When the media report cost ratio is 0.4.**

| **Time**  **(Day)** | **Probability of corporate disclosure** | **Probability of investor identification** | **Probability of media participation** |
| --- | --- | --- | --- |
| 1 | 0.30 | 0.30 | 0.40 |
| 2 | 0.29 | 0.38 | 0.43 |
| 3 | 0.28 | 0.42 | 0.45 |
| 4 | 0.27 | 0.45 | 0.46 |
| 5 | 0.27 | 0.47 | 0.47 |
| 6 | 0.26 | 0.48 | 0.47 |
| 7 | 0.26 | 0.49 | 0.48 |
| 8 | 0.25 | 0.50 | 0.48 |
| 9 | 0.25 | 0.51 | 0.48 |
| 10 | 0.25 | 0.51 | 0.49 |
| 11 | 0.24 | 0.52 | 0.49 |
| 12 | 0.24 | 0.53 | 0.49 |
| 13 | 0.24 | 0.53 | 0.49 |
| 14 | 0.23 | 0.53 | 0.50 |
| 15 | 0.23 | 0.54 | 0.50 |
| 16 | 0.23 | 0.54 | 0.50 |
| 17 | 0.23 | 0.54 | 0.50 |
| 18 | 0.22 | 0.55 | 0.50 |
| 19 | 0.22 | 0.55 | 0.50 |
| 20 | 0.22 | 0.55 | 0.51 |
| 21 | 0.22 | 0.55 | 0.51 |
| 22 | 0.22 | 0.56 | 0.51 |
| 23 | 0.21 | 0.56 | 0.51 |
| 24 | 0.21 | 0.56 | 0.51 |
| 25 | 0.21 | 0.56 | 0.51 |
| 26 | 0.21 | 0.56 | 0.51 |
| 27 | 0.21 | 0.56 | 0.51 |
| 28 | 0.21 | 0.56 | 0.51 |
| 29 | 0.20 | 0.57 | 0.52 |
| 30 | 0.20 | 0.57 | 0.52 |
| 31 | 0.20 | 0.57 | 0.52 |
| 32 | 0.20 | 0.57 | 0.52 |
| 33 | 0.20 | 0.57 | 0.52 |
| 34 | 0.20 | 0.57 | 0.52 |
| 35 | 0.20 | 0.57 | 0.52 |
| 36 | 0.19 | 0.57 | 0.52 |
| 37 | 0.19 | 0.57 | 0.52 |
| 38 | 0.19 | 0.57 | 0.52 |
| 39 | 0.19 | 0.57 | 0.52 |
| 40 | 0.19 | 0.58 | 0.52 |
| 41 | 0.19 | 0.58 | 0.52 |
| 42 | 0.19 | 0.58 | 0.52 |
| 43 | 0.19 | 0.58 | 0.52 |
| 44 | 0.19 | 0.58 | 0.52 |
| 45 | 0.18 | 0.58 | 0.53 |
| 46 | 0.18 | 0.58 | 0.53 |
| 47 | 0.18 | 0.58 | 0.53 |
| 48 | 0.18 | 0.58 | 0.53 |
| 49 | 0.18 | 0.58 | 0.53 |
| 50 | 0.18 | 0.58 | 0.53 |
